# “A Recipe for Disaster?”: Female-Breadwinner Relationships Threaten Heterosexual Scripts

**DOI:** 10.1007/s11199-025-01560-y

**Published:** 2025-02-19

**Authors:** Alexandra N. Fisher, Danu Anthony Stinson, Anastasija Kalajdzic, Hannah E. Dupuis, Erin E. Lowey, Elysia Desgrosseilliers, Annie MacIntosh

**Affiliations:** 1 Department of Psychology, School of Philosophy, Psychology and Language Sciences, University of Edinburgh, 7 George Square, Edinburgh, EH8 9JZ UK; 2 Department of Psychology, University of Victoria, Victoria, Canada

**Keywords:** Interpersonal relationships, Gender roles, Sex roles, Gender nonconforming, Relationship quality, Gender equality

## Abstract

Female breadwinner relationships (FBRs) occur when a woman earns more money than her male romantic partner. In four studies, we used diverse methods to document the threat that FBRs pose to heterosexual scripts (i.e., social conventions for heterosexual romance). First, a reflexive thematic analysis of 94 newspaper and magazine articles about FBRs identified themes concerning social stigma and feelings of gender threat (i.e., co-occurring feelings of gender nonconformity and inadequacy) that undermine well-being for FBRs, alongside themes concerning hope for a more egalitarian future. Next, two pre-registered experiments (*Ns* = 880 and 1612) revealed stigmatizing attitudes towards FBRs, which were perceived to be less desirable, worse quality, and less stable than male-breadwinner relationships. Finally, a cross-sectional study of married women and men (*N* = 511) affirmed that feelings of gender threat partially explained FBRs’ poor relationship outcomes. Across all four studies, and consistent with theories of fragile masculinity, men suffered worse gender threat than women in FBRs. These findings offer novel insight into heterosexual scripts and the punishing social consequences for people who violate those scripts and suggest that social stigma about FBRs may pose a barrier to gender equality in close relationships and in society.

**Supplementary Information:**

The online version contains supplementary material available at 10.1007/s11199-025-01560-y.

“*Are Female Breadwinners a Recipe for Disaster?*”

*- New York Post* (Stewart, 2014)

“*The Danger of Being a Breadwinning Wife*.”

*- The Telegraph* (Cavendish, 2015)

“*Female Breadwinners Pay a Cost for Career Success – Marital Stress*.”

*- The Globe and Mail* (Hansen, 2017)

In one of the best moments in the *Star Trek* movie franchise, the starship Enterprise travels back in time to 20th century Earth to avert planetary disaster. Hilarity ensues as the crew tries to make sense of antiquated cultural concepts like money, social inequality, and the punk aesthetic. We imagine that if the crew had encountered the newspaper headlines that are quoted above during their trip, they would have been equally perplexed to learn that the circumstance that warranted such dire warnings was in fact the mundane occurrence of a woman earning more money than her male romantic partner. We suggest that these headlines only make sense if the reader understands Western *heterosexual scripts*. Such scripts comprise mutually agreed upon social conventions that help people to make sense of romantic relationships between men and women by organizing sequences of behaviors into coherent stories (e.g., Cameron & Curry, 2020; Hoplock & Stinson, 2022; Laner & Ventrone, 2000; Vink et al., 2023 see also Baldwin, 1992; Cantor & Kihlstrom, 1985; Schank & Abelson, 1977; Simon & Gagnon, 1986).

Western heterosexual scripts have remained largely unchanged in the past 50 years, despite rapid social changes in other areas (Cameron & Curry, 2020; Rose, 2000), and dictate that women and men should occupy separate but complementary social roles (Fox & Murry, 2000). Men are expected to be agentic, dominant, and powerful in their romantic relationships with women (Good & Sanchez, 2010; Sanchez et al., 2012; Wang et al., 2013), all characteristics that support their prescribed role of provider within heterosexual relationships (Chaney et al., 2017). Women are expected to be communal, nurturing, and kind in their romantic relationships with men, all characteristics that support their prescribed role of caregiver within heterosexual relationships (Gardiner, 2002; Plummer, 2005). As such, while heterosexual scripts are nested within broader gender-role expectations for men and women, they also prescribe a narrower set of context-specific and gendered expectations for caregiving and provider behaviors within heterosexual relationships (Simon & Gagnon, 1986).

Although they pertain to romantic relationships, heterosexual scripts are also reinforced by organizational and workplace structures. Many organizations continue to be segregated along gendered lines such that women disproportionately occupy lower-ranking, lower paying positions while men predominate higher-ranking, higher paying positions (Carranza et al., 2023). Women also predominate in caregiving professions like healthcare and early education while men predominate agentic professions like science, technology, engineering, and mathematics (Croft et al., 2015). These disparities mirror heterosexual scripts and thus reinforce the expectation that women should serve as primary caregivers and men as primary breadwinners within their personal relationships.

Workplace policies, practices, and biases further codify heterosexual scripts. For example, the pervasive association of motherhood with caregiving and fatherhood with financial provision can manifest in discriminatory workplace practices (Bear & Glick, 2017; Berdahl & Moon, 2013). Mothers are often placed on the ‘mommy track,’ given roles that accommodate presumed caregiving responsibilities but can otherwise stifle career advancement. In contrast, fathers are often viewed as the primary providers for their families and, as such, are perceived as more dedicated and deserving of promotion and career advancement. These kinds of organizational norms and biases not only reinforce heterosexual scripts in the workplace but also sustain the economic and status disparities that uphold these scripts within men and women’s personal relationships.

Heterosexual scripts serve an informational function (e.g., “what happens on a first date?”), a predictive function (e.g., “how will he react when I offer to pay?”), and they serve as an aid to episodic memory (“how should I tell my proposal story?”; e.g., Berntsen & Rubin, 2004; Hoplock & Stinson, 2022). But they also serve a normative or prescriptive function by describing common and acceptable behavior for men and women within heterosexual romantic relationships, and within society more generally. Thus, adhering to heterosexual scripts offers a sense of predictability and ease to social interactions between men and women that can be comforting for heterosexual people (e.g., Day et al., 2011). When heterosexual men and women conform to their prescribed caregiver and provider roles within their romantic relationships, they create and affirm their own and one another’s gender through their actions (Butler, 1990; Morgenroth & Ryan, 2021). In turn, their gender-conforming actions garner a sense of legitimacy and social approval for their relationship, as society generally evaluates others more positively when they adhere to cultural scripts, including heterosexual scripts (e.g., Cast & Schweingruber, 2022).

But how do people react when heterosexual scripts are violated? A large body of feminist and queer scholarship has examined this question, offering compelling answers that elucidate both the punishing social reactions and the feelings of disorientation that people can suffer when they deviate from the often-compulsory dictates of heterosexual scripts; and conversely, the joy and fulfillment that many people find in nonconformity (e.g., Ahmed, 2006; Butler, 1990; Lamont, 2017; Morgenroth & Ryan, 2021; Rich, 1980). In the current research, we examine how people react when heterosexual scripts are violated from a novel angle by seeking to understand how people make sense of *female-breadwinner relationships* (FBRs), which are romantic relationships wherein a woman earns more than her male partner. By examining social, interpersonal, and intrapersonal reactions to these relationships from a social psychological perspective, we also hope to understand why men and women in FBRs may struggle (e.g., Coughlin & Wade, 2012).

## The Rise of the Female Breadwinner

In Western culture, a breadwinner is the person in a romantic relationship who works in the public sphere, earns the majority of household income, and contributes minimally to domestic work (e.g., childcare, housecleaning), whereas a homemaker is the person in a romantic relationship who is responsible for the majority of domestic work and may be considered the family’s secondary financial provider (e.g., Meisenbach, 2010). The heterosexual script specifies that men should fulfill the breadwinner role in their relationships with women, a role that has been deemed “a standard for male identity” (Meisenbach, 2010, p. 2), whereas women are expected to fulfill the homemaker role (Gaunt, 2013). However, this breadwinning script no longer reflects the modern reality. Due in part to the slow yet substantial impact of the second-wave feminist movement on Western women’s access to and success in the workplace since the 1970s, combined with changes in the workforce that devalue or even eliminate manual labour jobs that have been historically male-dominated (e.g., mining), fully 26% of Canadian women (ZipDo, 2024), 31% of women living in the United Kingdom (Inman, 2015), and 31–37% of women living in the United States hold the breadwinner title in their romantic relationships (Fox & Moyser, 2018; ZipDo, 2024). This statistic is only expected to increase in the future. This relatively rapid and ongoing social change in women’s breadwinning status provides a fertile opportunity to better understand the nature of heterosexual scripts by examining reactions to FBRs who violate the script and its breadwinning norm.

### Reactions to Female-Breadwinner Relationships

When people violate heterosexual scripts and behave in a way that is perceived to be inconsistent with their assigned gender role, such as when a woman earns more money than her male partner or a man engages in more domestic work and caregiving than his gender role prescribes, this gender nonconformity is noticed by observers and actors alike (e.g., Morgenroth & Ryan, 2021). Breadwinning is a valued, high-status role that is a central component of the heterosexual male gender role (Cunningham, 2008; England, 2010). As a result, high-status breadwinning women and their male partners contravene gender norms. Thus, we propose that FBRs will be stigmatized because their gender nonconformity threatens heterosexual scripts and the binary system of gender/sex they represent, a punishing response that is ultimately aimed at forcing script violators back into compliance (see Morgenroth & Ryan, 2021).

To date, these dynamics have not been applied to understand the experiences of FBRs. There is evidence that working mothers, including breadwinner mothers, and stay-at-home fathers are evaluated more harshly than their counterparts who conform to the heterosexual script (Brescoll & Uhlmann, 2005). Similarly, women who violate gender-role expectations in the workplace by behaving agentically or by occupying positions of authority are often evaluated negatively (Brescoll et al., 2018). Such women are also accused of lacking concern for social order and disrespecting traditional relationships, and as a result, people often respond to such women with moral outrage in the form of disgust or contempt. Men and women who violate heterosexual scripts in the workplace experience greater harassment and mistreatment than their script-conforming counterparts (Berdahl & Moon, 2013). First-person accounts of female breadwinning also suggest that breadwinning women often feel stigmatized (e.g., Chesley, 2017). Thus, it is reasonable to propose that men and women who participate in FBRs will be subjected to similar social punishments for violating heterosexual scripts. Specifically, we expect that stigma about FBRs will include the assumption that they are poor quality relationships with a higher risk of divorce than their counterparts who conform to the breadwinner script. Men and women who take part in FBRs may also be derogated and pathologized for their choice.

### Consequences for FBRs

#### Poor Relationship Quality

We anticipate that men and women in FBRs will experience worse relationship quality than their script-conforming peers. There is a growing body of research suggesting that this is the case (Bertrand et al., 2015; Coughlin & Wade, 2012; Furdyna et al., 2008; Pierce et al., 2013; Syrda, 2020; Tichenor, 1999; Wilcox & Nock, 2006). Men in relationships with breadwinning women have lower relationship satisfaction than men in relationships where they are the breadwinner (Pierce et al., 2013; Syrda, 2020). Breadwinning women, in contrast, do not always report lower relationship satisfaction (Rogers & DeBoer, 2001), but highly successful women report worse relationship quality when they achieve higher personal status than their male partner (Vink et al., 2023). Some of this difference in relationship outcomes between FBRs and their script-conforming counterparts is likely due to the stress of being subjected to social stigma. However, we suggest men and women in FBRs may also struggle because they experience gender threat.

#### Gender Threat

We suggest that the combined feelings of gender nonconformity and inadequacy that can accompany heterosexual script violations – which together comprise gender threat – help to explain the poor relationship outcomes for FBRs. Plenty of people are perfectly happy with their gender nonconformity and welcome the *gender trouble* it causes by challenging, or disrupting, the reinforcing cycle of the gender/sex binary (Butler, 1990; Morgenroth & Ryan, 2021). Indeed, the American Psychological Association and Canadian Psychological Association are very clear that gender nonconformity is normal and natural (American Psychological Association, 2015; Cameron & Stinson, 2022), and we absolutely concur. Many gender troublemakers who are members of the LGBTQ + community embrace their gender nonconformity in part by rejecting heterosexual scripts and building new, queer interpersonal scripts (e.g., Lamont, 2017; Rose, 2000; see also Ahmed, 2006). Some breadwinning wives also push back against heterosexual scripts when making sense of their breadwinning role by envisioning the benefits of their contributions and their role in the family independent of prescribed gender norms – or despite them (Medved, 2016; Meisenbach, 2010).

However, for a variety of possible reasons, men and women who participate in heterosexual relationships may not reject heterosexual norms, including heterosexual scripts. For example, they may experience reduced motivation to engage in gender troublemaking because of the privileges they enjoy through attainable conformity, they may lack role models for such troublemaking, or they may experience heightened pressure from a norm-abiding partner. As a result, men and women in FBRs may internalize the stigma that they are subjected to because they violate heterosexual scripts. Thus, they may judge themselves negatively for their gender nonconformity, leading to feelings of inadequacy. Indeed, prior research suggests that men experience poor mental and physical health when they are married to a breadwinner wife, as evidenced by greater medication usage for erectile dysfunction, anxiety, and depression (Pierce et al., 2013; Syrda, 2020). Researchers presume that such negative effects occur because of threatened masculinity.

Breadwinning women in relationships with men also experience an internal sense of conflict and guilt about being the breadwinner, presumably for failing in their roles of wife and mother (Meisenbach, 2010). In turn, experiencing gender threat (i.e., heightened feelings of inadequacy stemming from perceived gender nonconformity) may help to explain why FBRs suffer lower relationship quality. People who feel inadequate doubt their value as relational partners, leading them to engage in self-protective behaviors that can undermine the quality of their relationships via a process of behavioral confirmation (e.g., Stinson et al., 2009; for a review see Cameron & Granger, 2019). Thus, we propose that gender threat will help to explain why FBRs experience worse relationship quality than their counterparts in male-breadwinner relationships (MBRs).

#### Precarious Masculinity

We also predict that gender threat will be worse for men in FBRs than women (see also Morgenroth & Ryan, 2021). Manhood is notoriously precarious, such that men must continually act to prove their masculinity and validate their manhood (Bosson & Vandello, 2011; Vandello & Bosson, 2013; Vandello et al., 2008). Womanhood, in contrast, is seen as a relatively more stable state that is less easily threatened (Gardiner, 2002; Plummer, 2005). Thus, we predict that men’s participation in an FBR more readily impacts evaluations of their gender nonconformity, relative to the effect of women’s breadwinning status on evaluations of their gender nonconformity. Further, compared to women, we expect that violating the breadwinner script will have a greater impact on men’s feelings of inadequacy and relationship quality. Because of these gender effects, we also anticipate that gender threat (i.e., heightened feelings of inadequacy stemming from perceived gender nonconformity) will explain a larger proportion of the association between breadwinner status and relationship quality for men than for women.

### The Current Research

In four studies, we use diverse qualitative and quantitative methods to document social, interpersonal, and intrapersonal reactions to FBRs. Our mixed-methods approach allows us to gain more rich and credible scientific evidence than could be gained by any one method on its own, because our approach allows us to triangulate evidence across multiple methods that have unique and non-overlapping strengths and weaknesses (Diener et al., 2022). Our research is also timely given the rapidly increasing prevalence of FBRs around the globe, and the pressing need to understand the stressors that affect personal and relational well-being for these couples.

Because the media plays a central role in communicating both the content of cultural scripts and the social consequences of script violations (e.g., Bandura, 1986), in Study 1 we conducted a reflexive thematic analysis of 94 magazine and newspaper articles about female breadwinners (following Braun & Clarke, 2022). Our analysis sought to understand how the magazine and newspaper articles portrayed heterosexual scripts by describing the roles and behaviors that are expected and acceptable within romantic relationships between men and women; and conversely, how they portrayed FBRs that cause gender trouble by violating those heterosexual scripts. We were particularly interested in understanding how the articles portrayed gender identity, gender roles, and whether they described gender threat (i.e., heightened feelings of inadequacy stemming from perceived gender nonconformity) within FBRs. This analysis offers a rich and compelling picture of the social context that FBRs are embedded within, allowing us to assess the perceived threat that FBRs pose to heterosexual scripts and the cultural response to FBRs as gender troublemakers. First-person accounts within the articles may also speak to our hypotheses concerning feelings of gender threat and relationship quality.

We also conducted two pre-registered experiments and one survey study that sought to test our confirmatory hypotheses concerning the psychological consequences of violating heterosexual scripts by participating in an FBR. Studies 2a and 2b (total *N* = 2143) asked participants to evaluate the relationship quality and gender threat (i.e., gender nonconformity and inadequacy) experienced by a man and woman in an FBR or a male-breadwinner relationship (MBR). People often rely on implicit knowledge like social scripts to evaluate others’ psychology when they lack individuating information (e.g., Rubinstein et al., 2018). Furthermore, social scripts include the consequences of script violations (e.g., “if he wears a dress, he will be rejected and feel ashamed”). Thus, in our experiments we predicted that compared to the male breadwinner couple, participants would perceive that the female breadwinner couple experiences worse relationship quality and more gender threat (i.e., stronger feelings of gender nonconformity and inadequacy); we also anticipate that these effects would be greater for evaluations of men than women.

In Study 3 (*N* = 511) we surveyed married adults in heterosexual relationships and examined the associations among breadwinning status, gender threat (i.e., feelings of gender nonconformity and inadequacy), and relationship quality. Once again, we predicted that relative to the participants who were in MBRs, participants in FBRs would experience worse relationship quality and more gender threat, and heightened gender threat would explain, in part, the worse relationship outcomes for FBRs. Further, we expected that all these predicted effects would be stronger for men than for women.

## Study 1: The Breadwinner Script and Magazine and Newspaper Articles

Media reflects, shapes, and reinforces cultural values and norms, including heterosexual scripts (e.g., Bandura, 1986; Galdi et al., 2014; Keller, 2011). Thus, we sought to understand how newspaper and magazine articles about female breadwinners portrayed the roles and behaviors that are expected and acceptable within romantic relationships between men and women; and conversely, how the newspaper and magazine articles portrayed FBRs who cause gender trouble by violating those heterosexual scripts. We were particularly interested in understanding how the media portrayed gender identity, gender roles, gender threat (i.e., feelings of gender nonconformity and inadequacy), and power dynamics within FBRs. We used reflexive thematic analysis to answer these research questions, which allowed us to identify, analyze, and report patterns of shared meaning, or themes, that we derived from our sampling of media (Braun & Clarke, 2006, 2019, 2022). We adopted a critical realist perspective in our analysis, which means that we assumed that individuals make meaning from their experiences, that social context (including media) shapes the meanings people make, and that material reality informs both of those factors.

Reflexive thematic analysis is nested within a critical psychology tradition that emphasizes researcher subjectivity as a resource, rather than a source of bias, and considers meaning and knowledge as partial, situated, and contextual (e.g., Braun & Clarke, 2022). In accordance with this epistemology, themes are constructed through researcher engagement with the data, mediated by the research values and experience that the researcher brings to the process (i.e., positionality). We have already detailed the theoretical and epistemological positions that informed our analysis; some of our research team’s lived experience as female breadwinners and gender troublemakers was a further source of knowledge in our analysis. Critical approaches also aim to interrogate systems of power when seeking to understand socially embedded patterns of meaning, making it a good fit for the current research, which examines, in part, the power hierarchy that exists between women and men.

### Method

We consulted with our ethics board at the University of Victoria, Canada, prior to project commencement. Data were collected as part of a master’s thesis concerning portrayals of FBRs in the media (Kalajdzic, 2020), but the coding and analyses were conducted from scratch for the current research.

#### Data Collection

We restricted our search to articles from news sources in Canada, the United Kingdom, and the United States because our research questions are culturally situated, and these three English-speaking countries share cultural values and have a similar proportion of female breadwinners in their populations (Cory & Stirling, 2015; Fox & Moyser, 2018; Wang et al., 2013). We also restricted the article search to the years 2013–2019, inclusive, in part to limit our sample to a manageable size and in part because 2013 marked a notable spike in popularity for the phrase “female breadwinner” in news content (Google, n.d.). Data were collected in early 2019.

Within these parameters, three research assistants (RAs) searched for magazine and newspaper articles on *Access World News* – an online newspaper and magazine database that was accessible through our institution’s library – containing the phrase “female breadwinner,” which yielded the best results in our pilot searches in terms of article volume and relevance. RAs included an article in their sample if the title, brief article description, and/or a brief skim of the content suggested that it fit our search criteria and was primarily about female breadwinners. One of the RAs also searched Google using the same search term and identified articles that fit our inclusion criteria, were not behind a paywall, and were not already sampled from *Access World News.* This initial search yielded 246 articles. Next, AK read each article to determine whether it truly focused on female breadwinners (defined as a woman earning most of the income in her relationship) and concerned the cultures of interest. She also excluded articles if: (1) the article only concerned single mothers as breadwinners (because we were interested in couple scripts); (2) the article only concerned high-earning women with no mention of their romantic relationship; (3) the article mentioned female breadwinners in passing with no relation to the rest of the article content; (4) the article was a re-print of an article we already sampled. This evaluation resulted in a final sample size of 94 relevant articles. A numbered list of articles and article descriptives are presented in the Online Materials (OM) available on the Open Science Framework (OSF): https://osf.io/tuyj5/.

#### Reflexive Thematic Analysis

We followed the analytic procedure outlined by Braun and Clarke (2022). To support methodological integrity, each stage of the analytic process was documented with numbered data files and thematic maps to ensure that the development of themes was clear and traceable.

First, DAS and EEL independently read a sampling of 30 articles on multiple occasions, considered what was written (i.e., who was described, cited, and quoted in the article; what was said about the breadwinner and her partner; how were FBRs described; and most importantly, how were gender identity, roles, power dynamics, and threat portrayed in the articles), and assigned the observations to preliminary codes using the MAXQDA 2022 qualitative analysis software (VERBI Software, 2021). They also kept memos to keep track of their thoughts throughout the study process. Next, the coders met to discuss and refine their preliminary codes and then EEL coded the remaining articles. Then DAS reviewed and refined the codes once more before developing themes. This process was primarily deductive, in that DAS used theories concerning gender identity, gender threat, and social scripts (as outlined in the general introduction) to inform the creation of themes. However, she was also open to discover novel patterns of meaning in the data.

Furthermore, DAS sought to capture both semantic meanings that were explicit in the media, and latent meanings that were implicit or unspoken but understood through careful consideration of contextual cues and her own cultural knowledge and lived experience as a female breadwinner and gender troublemaker. She then collated codes into initial themes and sub-themes and reviewed and refined the themes by probing whether the core concepts or meanings expressed by the themes spoke to the research questions; ANF also aided in this review and refinement process by providing a “fresh” perspective on the themes and their relation to our research questions. Finally, DAS wrote the analysis, further refining and renaming the themes as needed during the writing and revision process, for clarity and conciseness. At this point, if it appeared that a particular theme was relevant to our a priori hypotheses, this was noted in the analysis.

### Results

**Fig. 1:**
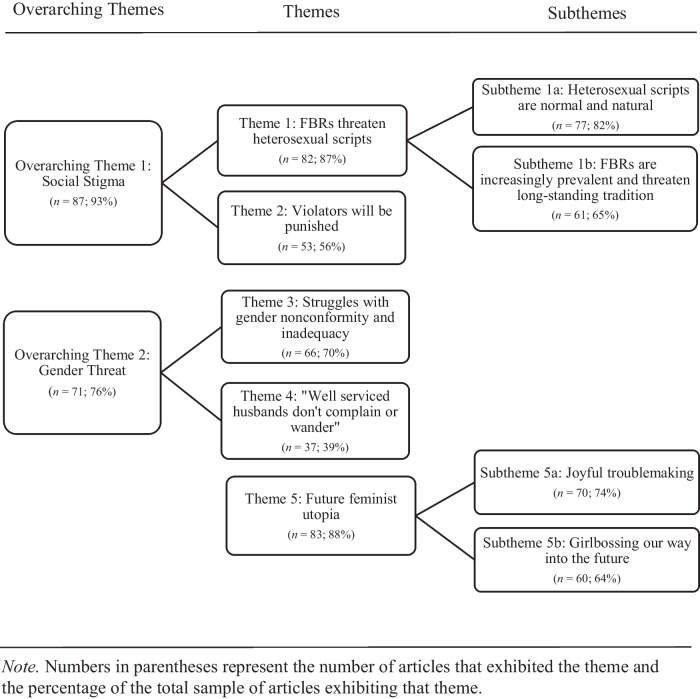
Map depicting the relation among overarching themes, themes, and subthemes in Study 1

Figure 1 depicts the relation among overarching themes, themes, and sub-themes that comprised our analysis, and Table 1 provides example data extracts for each theme/subtheme.

**Table 1:** Example Data Extracts for Themes and Sub-Themes in Study 1

Overarching theme	Theme/Subtheme	Example data extracts
Social Stigma	Theme 1: FBRs threaten heterosexual scripts	- [The] data reveals a revolution in the traditional roles of men and women that extends from college campuses to the workplace to the neighborhoods across this nation. (A4)
	Subtheme 1a: Heterosexual scripts are normal and natural	- …this feeling just came out of some deep place that one should be able to support a wife if one is going to be a husband. (A36) - ‘…homemaker was the proper role for a Southern girl.’ (A4)
	Subtheme 1b: FBRs are increasingly prevalent and threaten long-standing tradition	- This change is just another milestone in the dramatic transformation we have seen in family structure and family dynamics over the past 50 years or so. (A65) - …the myths of the traditional household are shattering… (A80) - Those most concerned seemed fearful that American society may be on the verge of becoming a matriarchy. (A90)
	Theme 2: Violators will be punished	- This has created a real backlash for the women who have taken this path… (A10) - ‘I have been punished for being a successful woman in a way I never expected.’ (A28) - ‘Is it worth it to have the man be the second highest paid person in the house?’ Once again, the point was raised that husbands are more likely to cheat when wives are earning more. (A43)
Gender Threat	Theme 3: Struggles with gender nonconformity and inadequacy	- ‘…I get insecure as being seen as an ice-cold workaholic mom…’ (A3) - Few women are happy married to a freeloader. (A16) - ‘There is some pressure from the outside world to live a certain way or you are inadequate.’ (A62) - The growing role of working women has often been blamed for making men feel sidelined, emasculated and unsure of their role in the family. (A75)
	Theme 4: “Well serviced husbands don’t complain or wander”	- ‘If you want to work, fine, but you can’t skirt these traditional responsibilities at home.’ (A7) - …you have to be careful you’re not shaming him or making him feel unworthy. (A84) - …the [breadwinning] woman often felt like she had to play down her own economic contributions to the household while offering her husband reassurances that she valued his masculinity. (A1)
	Theme 5: Future Feminist Utopia	- ‘Maybe in the future this will just be the norm, and it won’t be unusual to anybody…’ (A46)
	Subtheme 5a: Joyful troublemaking	- ‘If they are open-minded and adjust their roles accordingly… then things generally work out just fine.’ (A36). - Female breadwinners become invaluable role models for both their daughters and sons. (A57) - The same study found that the more women contribute to the household income, the happier they are. (A87)
	Subtheme 5b: Girlbossing our way into the future	- Until society adapts, it seems female breadwinners will have to find out the answers for themselves. (A45) - ‘Sure there is still plenty of work to be done in terms of closing the wage and wealth gaps, but becoming highly educated and ‘bringing home the bacon’ can only help our journey towards new progress in these areas’ (A14)

#### Overarching Theme 1: Social Stigma

Nearly every article in our sample communicated that heterosexual scripts were normal and natural for romantic relationships between men and women, and if couples violated that script by participating in an FBR, they would experience punishing social stigma (see Table 1 for example data extracts for this overarching theme and its constituent themes and subthemes). This overarching theme speaks to our theorizing concerning the stigmatizing social response to FBRs as gender troublemakers.

***Theme 1: FBRs Threaten Heterosexual Scripts***

Nearly every article communicated that FBRs represent a dramatic and recent social change that threatens heterosexual scripts and the normal and natural relations between men and women that they represent (Theme 1). The articles made this point implicitly and explicitly. First, articles described heterosexual scripts as normal and natural and desirable (Subtheme 1a). For example, one article opined that “everyone is better off if the man is the achiever outside the home while the woman takes care of domestic duties” (A7). This pervasive endorsement of MBRs in articles about FBRs was surprising but telling. People are often strongly motivated to defend and uphold their treasured cultural institutions and values when they perceive a threat to those institutions and values, a defensive reaction that can help to sooth the anxiety that such threats engender (e.g., Jost & Hunyady, 2003). Thus, the articles’ endorsement and defense of MBRs may reflect a system justifying reaction that indirectly reveals the threat that FBRs pose to heterosexual scripts.

The articles also directly stated that FBRs threaten heterosexual scripts (Subtheme 1b). Consistent with research and theorizing concerning the characteristics of the *moral panic* response that societies can exhibit towards groups that are perceived to threaten important cultural values and norms (e.g., Goode & Ben-Yehuda, 1994), the articles characterized FBRs as a social group that is relatively new, increasing in prevalence, and a threat to long-held social traditions, specifically, heterosexual scripts (see Kalajdzic, 2020). In making this point, the articles sometimes used the 1950s and 1960s as a point of comparison for the unwelcome social changes heralded by FBRs, which may have reflected nostalgia for a period in Western history when gender roles were clearly prescribed and enforced. As one article put it, the changing gender norms that are heralded by the FBR “hit the nation like of a ton of bricks, crashing down on the *Leave It to Beaver* and *Father Knows Best* image of the American nuclear family” (A6). The hyperbolic imagery and language that was used in this quote and in many of the other articles’ descriptions of FBRs was also consistent with a moral panic response (e.g., “dramatic transformation,” “revolution,” “shattering,” “matriarchy;” see Table 1) and served to emphasize the magnitude of the gender trouble that these relationships were perceived to cause by challenging and disrupting the gender/sex binary as expressed in heterosexual scripts.

***Theme 2: Violators Will Be Punished***

The articles often described widespread social condemnation of FBRs. As one article stated, “The feedback [FBRs] receive from the culture is clear: Men should be earning more so that they can provide for their families, and if they don’t, it’s symptomatic of a problem” (A23). This kind of social condemnation against a group that is perceived to cause gender trouble by disrupting the gender/sex binary is a common method of social control, because it increases the costs of violating heterosexual scripts, thereby motivating offenders to conform to avoid said costs (Morgenroth & Ryan, 2021).

The articles commonly used cautionary tales to illustrate how female breadwinners, in particular, would be punished for violating heterosexual scripts. Cautionary tales are social narratives that are often used as folk teaching devices concerning the negative consequences of failing to abide by common social norms or values (think, Aesop’s fables; e.g., Marett, 2014). In such tales, the protagonist typically suffers a terrible outcome because they foolishly failed to conform to a desired social attitude or behavior, and thus audiences often blame the protagonist for their negative outcomes. In the articles we sampled, cautionary tales about FBRs typically began by describing the life of a female breadwinner who was flying high on her career success with an apparently supportive partner, until she discovered that her partner was actually resentful and her marriage was crumbling. Divorce and infidelity were the most common punishments for female breadwinners in these stories. For example, one article asked, “Marriages could break up. Is it worth it? Is making a substantial salary worth losing your husband?” (A43). By using this narrative device, the articles created the impression that breadwinner women’s gender-nonconforming ambition and pursuit of career success was foolish and hubristic, and hence they ultimately deserved the infidelity or divorce they suffered.

#### Overarching Theme 2: Gender Threat

Most articles portrayed the men and women in FBRs as struggling immensely with gender nonconformity and feelings of failure and inadequacy, which together comprise gender threat (see Table 1). The articles also suggested women should return to the “primrose path” (A28) of enacting the female role in heterosexual scripts to resolve those problems. This overarching theme is relevant to our hypotheses because it illustrates how people in FBRs, and especially men in FBRs, are perceived to experience gender threat more commonly than their MBR counterparts. It also illustrates attempts to make FBRs conform to heterosexual scripts.

***Theme 3: Struggles with Gender Nonconformity and Inadequacy***

The articles frequently described the gender nonconforming behavior of women and men in FBRs, often framed in terms of the male gender role. Thus, women’s gender nonconformity was illustrated by emphasizing their ambition and career drive, key masculine characteristics, and with nicknames like “alpha female” (A10) and “alpha wife” (A63) that emphasized their dominance. In contrast, men’s gender nonconformity was illustrated by portraying them as lazy freeloaders who lacked the ambition that was required to be a proper man. As one article asked: “Have millennial men defeated gender stereotypes to embrace a new working world of equality? Or could it be that they are just really, really lazy?” (A87). This focus on the male gender role when describing FBRs’ gender nonconformity may reflect the centrality of breadwinning to both masculinity and the specifics of FBRs’ gender nonconformity, or it may reflect androcentrism, which is a cultural practice of centering men and masculinity in social cognition (Bailey et al., 2019).

The articles also linked FBRs’ gender nonconformity to feelings of inadequacy. The articles described breadwinning women’s inadequacy at performing their roles as wives and mothers, which was framed as a consequence of their selfish attempts to “have it all” (A50). Men in FBRs were also deemed to be inadequate at fulfilling male role expectancies. Consistent with theories of precarious masculinity, which argue that manhood must be constantly earned and proven (e.g., Vandello & Bosson, 2013), the articles also suggested that having a breadwinner wife was emasculating. In this way, men were often framed as victims of their wives’ career aspirations. This sentiment, and the core message of this theme is encapsulated by one article’s (somewhat ironic) suggestion that breadwinner wives “are associated with the image of alpha female, emasculator of men, shirker of motherly and homemaking responsibilities” (A10).

***Theme 4: “Well Serviced Husbands Don’t Complain or Wander”***

The articles often suggested that female breadwinners should resolve their husband’s gender threat by enacting heterosexual scripts. Female breadwinners in the articles seemed to be taking this advice to heart, as many articles discussed the so-called “second shift” or “double duty” that female breadwinners do at home, taking care of domestic work after a long day of working outside the home (see Chaney et al., 2017; Tichenor, 1999; but see also Brailey & Slatton, 2019, who argue that the concept of “second shift” is a white cultural construct). Consistent with research suggesting that women are socialized to protect and bolster men’s egos (Impett & Peplau, 2003), the articles also urged female breadwinners to prioritize their husband’s emotional needs and protect his masculinity. Other tips for breadwinning wives who needed to sooth their husband’s threatened masculinity included “letting men pay the bill at the restaurant” (A19), leaving the house without a wallet or keys so their husband has “an opportunity to be the provider” (A45), allowing their husband to pay for “a particular family need, creating a sense of purpose around that income” (A57), downplaying their career success and income (e.g., A17), and providing frequent sex, because, “Husbands who are well serviced don’t complain or wander” (A63). Thus, the articles explicitly urged breadwinning women to conform to heterosexual scripts to solve the problems their gender troublemaking caused.

***Theme 5: Future Feminist Utopia***

Many articles celebrated female breadwinning and envisioned a future where it would be considered normal within society for women to be the breadwinner (Theme 5; see Table 1). Because of this, a palpable tension existed in most articles, whereby the stigmatizing portrayals of female breadwinners and descriptions of the gender threat those relationships posed often co-existed with upbeat and even joyful stories of successful FBRs (Subtheme 5a). For example, one article asserted that “couples who divide household chores and child-rearing jobs report having the best marriage and sexual satisfaction” (A51). Successful FBRs typically negotiated egalitarian interpersonal scripts for their relationships that did not depend on heterosexual scripts (see also, Medved, 2016). A similar practice is often observed among sexual and gender minority couples (Rose, 2000; see also Simon & Gagnon, 1986). Such couples usually decide how domestic and childcare work is distributed, who is responsible for paying bills, and who has decision-making power – all common components of heterosexual scripts – based on individual and dyadic preferences rather than gender/sex or gender-roles (see Rostosky & Riggle, 2017). Thus, the adoption of egalitarian interpersonal scripts may be a key component of FBRs’ gender troublemaking, as the practice eschews heterosexual norms concerning domestic work and power, and it may also represent a key component of relationship success for many couples.

The articles also included predictions about a utopic feminist future where egalitarianism becomes the “new normal” (A8), but they acknowledged that society needed to play cultural catch-up to get there (Subtheme 5b). As one article put it, “Female higher-earners interviewed for this article almost uniformly expressed frustration with their sense that society has not quite caught up with them” (A45). The articles cited numerous barriers to egalitarian progress, including men’s unwillingness to participate in FBRs -- the type of man who was willing to marry a breadwinning woman was characterized as a “rare unicorn” (A41) – and the need for government and workplace policies that would allow women to step away from the unpaid domestic labour they currently perform to keep society functioning (e.g., Fisher & Ryan, 2021; Fisher et al., 2024). Rather than encouraging gender troublemaking and collective action to overcome these barriers, most articles espoused a version of neoliberal feminism that emphasized individual women’s agency and ability to overcome barriers to success (e.g., “girl power” and “lean in” messaging is of this ilk; see Keller, 2011). They frequently used empowerment messaging to optimistically argue that women can individually overcome any barriers that they may face in their quest to achieve equality. As one article asserted, “There is a lot we haven’t asked for in our rise as breadwinners and matriarchs. So ask for that pay raise and leadership role at work and ask for help with housework and caring for the children. Empower your voice to unequivocally help tip the scales towards better balance at home and equality at work” (A14). Unfortunately, when viewed through a critical feminist lens, these assertions can seem like a soothing fantasy that ultimately supports the status quo by burdening women with the task of overcoming their own oppression (e.g., Desgrosseilliers & Stinson, 2025; Kim et al., 2018).

### Discussion

Our reflexive thematic analysis of magazine and newspaper articles provided a nuanced picture of the social stigma surrounding FBRs who violate heterosexual scripts and illustrates the coercive attempts to bring such couples back to what Ahmed (2006) calls “the straight line of heterosexuality” (p. 78). FBRs were stigmatized in the media because they violate heterosexual scripts and were thus condemned to suffer relationship dissatisfaction and divorce because of their nonconformity. Newspaper and magazine portrayals of FBRs also suggested that participants in such relationships would experience gender threat, in the form of feelings of gender nonconformity and feelings of inadequacy. Men were portrayed as feeling emasculated and demoralized by higher earning wives. Ultimately, these feelings of gender threat were blamed for undermining the quality of female breadwinner relationships. Yet, these very same articles also offered a convenient remedy for these poor outcomes by suggesting that if breadwinning women would simply conform to heterosexual scripts, their relationships would improve. Taken together, these popular portrayals of FBRs likely serve to preserve heterosexual scripts by dissuading the public from violating them in the first place. The articles also explained how some couples can fare well in FBRs if they embrace egalitarian norms and reject heteronormative scripts for their relationships. Such couples may have managed to resist internalizing the social stigma concerning FBRs, and thus they did not experience their gender nonconformity as threatening. Our next studies use quantitative methods to examine these inter- and intrapersonal consequences of heterosexual script violations in greater detail.

## Studies 2a & 2b: The Breadwinner Script and Judgements of Others

Next, we assessed people’s implicit knowledge concerning the consequences of violating heterosexual scripts. In two pre-registered experiments, we used a common social-psychological method for assessing implicit knowledge like heterosexual scripts, including contingencies concerning script violations (e.g., Hoplock et al., 2019): We asked participants to evaluate the relationship quality and gender threat experienced by a hypothetical man and woman in an FBR or an MBR. Participants’ responses should reflect their implicit contingencies about heterosexual script violations. Thus, we predicted that compared to the male breadwinner couple, participants would perceive that the female breadwinner couple experiences worse relationship quality and more gender threat (i.e., greater feelings of gender nonconformity and inadequacy). We also anticipated that these effects would be stronger for evaluations of men than women. Because Study 2b replicated Study 2a, we describe both studies together.

### Method

In both experiments, we describe how we determined our sample size, all data exclusions, all manipulations, and most study measures (additional measures, pre-registrations, and analyses for each experiment are described in the OM: https://osf.io/tuyj5/). This research received approval from the Human Research Ethics Office at the University of Victoria, Canada (approval number: 17–166). Data were collected in June 2017 (Study 2a) and March 2019 (Study 2b). We neglected to obtain approval from participants to share their data online (it was not common practice at the time), but data was made available to reviewers and it can be requested by directly contacting the corresponding author. Study materials and analytic code are available in the OM (https://osf.io/tuyj5).

#### Power Analyses and Sample Size

A priori power analyses using G*Power (Faul et al., 2007) indicated that to detect a small interaction effect of *f*^2^ = 0.10 with 80% power and α = 0.05, we would need a sample of approximately 800 people. Thus, for Study 2a, we aimed to recruit 950 participants to account for exclusions. For Study 2b, given the small to medium effect sizes observed in Study 2a and recommended practices for sample size planning for replication studies (Anderson & Maxwell, 2017; Schönbrodt & Perugini, 2013), we aimed to collect a sample of 1600 participants to account for possible over-inflation of our previously observed effect sizes and possible exclusions. A sample of this size with α = 0.05 would allow us to detect a small interaction effect of *f*^2^ = 0.10 with 98% power.

#### Participants

For both Studies, participants were recruited through Amazon’s Mechanical Turk (MTurk) and needed to be adults (age 18+) living in Canada or the United States. They were paid $0.30 in appreciation for their time.

***Study 2a***

The original sample size of 1022 participants was slightly larger than the planned sample size due to a researcher error when launching the survey on MTurk (we failed to account for participants in a test launch of the survey when setting the desired sample size). We excluded 121 participants (11.8%) for the following reasons: submission from a duplicate IP address (*n* = 24); responding negatively to the item “I tried to answer the questions honestly” (*n* = 3); failing one or more of the attention check questions (e.g., “for validation purposes, select E as a response;” *n* = 73); completed less than 50% of the measures (*n* = 21). This resulted in a final sample of 901 participants (*M*_age_ = 35.8 years, *SD*_age_ = 11.8 years) who identified as women (*n* = 560, 62.2% ), men (*n* = 333, 37.0%), nonbinary (*n* = 1, 0.1%), or declined to answer (*n* = 7, 0.8%). Furthermore, participants identified as Heterosexual (*n* = 777, 86.2%), LGBTQ+ (*n* = 120, 13.3%), or declined to answer (*n* = 4, 0.4%). Finally, participants identified as Black/African American (*n* = 73, 8.1%), East Asian/Southeast Asian (*n* = 58, 6.4%), East Indian or South Asian (*n* = 12, 1.3%), Latino/Latina/Latinx (*n* = 54, 6.0%), Multiracial (*n* = 16, 1.8%), Native American/Native Hawaiian/other Pacific Islander (*n* = 5, 0.6%), White/Caucasian (*n* = 625, 69.4%), or another race/ethnicity (*n* = 58, 6.4%).

***Study 2b***

The original sample size was 1616, but we excluded 394 participants (24.4%) for the following reasons: submission from a duplicate IP address (*n* = 101); responding negatively to the item “I tried to answer the questions honestly” (*n* = 97); failing one or more of the attention check questions (e.g., “Please select “moderately disagree” for this statement;” *n* = 196). This resulted in a final sample of 1222 participants (*M*_age_ = 37.25 years, *SD*_age_ = 12.65 years). Participants identified as women (*n* = 731, 59.8% women), men (*n* = 479, 39.2%), nonbinary (*n* = 3, 0.3%), or they declined to answer (*n* = 9, 0.7%). In terms of sexual orientation, participants identified as heterosexual (*n* = 1044, 85.4%) and LGBTQ+ (*n* = 178, 14.6%). Finally, they identified as Black/African American (*n* = 98, 8%), East Asian or Southeast Asian (*n* = 89, 7.3%), East Indian or South Asian (*n* = 11, 0.9%), Latino/Latina/Latinx (*n* = 58, 4.7%), Native American/Native Hawaiian/other Pacific Islander (*n* = 12, 1.0%), Multiracial (*n* = 14, 1.1%), White/Caucasian (*n* = 865, 70.8%), and another race/ethnicity (*n* = 75, 6.1%).

#### Procedure and Design

The procedure was identical for both studies. Potential participants were invited to take a survey about perceptions of relationships. Interested participants were directed to a Qualtrics survey where they first read the implied consent and then completed a brief demographic survey (e.g., age, sexual orientation) and measures of personality and attitudes (e.g., gender attitudes). Participants then read the following vignette describing a fictional relationship between a married heterosexual couple, Olivia and Zach, who met a few years ago “when they were both hired for the same position at their company.” The vignette described how one partner recently received a raise so that they now earn “significantly more money” than their spouse. The vignette also included details about the couple’s hobbies and leisure preferences:

Zach and Olivia have been together for some time now. They met when they were both hired for the same position at their company and it wasn’t long before their workplace flirtation led them to start dating. Now they are married and share an apartment. Both Zach and Olivia still work for the same company. A few months ago Olivia (Zach) received a raise and now earns significantly more money than Zach (Olivia). When they aren’t busy with work, Olivia and Zach like to entertain friends at their apartment, go out to see live music, or daytrip to the local beach.

Both studies employed a 2 (Relationship Type: MBR vs. FBR) x 2 (Target: Olivia vs. Zach) factorial design. Participants were randomly assigned to one of two breadwinner conditions: Participants in the *MBR* condition learned that Zach earned the raise whereas participants in the *FBR* condition learned that Olivia earned the raise. Further, participants were randomly assigned to one of two target conditions, in which they answered questions about either Zach’s or Olivia’s (i.e., the *target*) relational outcomes. Finally, participants were debriefed, thanked for their time, and awarded their compensation.

#### Measures

Items were phrased to match the gender/sex of the target. Measures were the same for both studies, but two additional measures were added to Study 2b, as indicated below.

***Relationship Quality***

Participants used the same 7-point scale to answer five questions assessing the target’s relationship satisfaction (e.g., “She would feel satisfied in this relationship”), three questions assessing the target’s commitment (e.g., “He would be committed to maintaining this relationship”), and four items assessing the target’s overall relationship quality (“She would avoid being vulnerable with her partner”). Items were coded so that higher scores reflected more positive relationship quality, then averaged (Study 2a α = 0.92; Study 2b α = 0.91). Items were adapted from similar measures members of our lab used in previous research (e.g., Fisher et al., 2021) and based on theory and research concerning attachment security (Cameron et al., 2012), satisfaction and commitment (Rusbult, 1980), and perceived regard (Gaucher et al., 2012).

***Relationship Dissolution***

In Study 2b only, participants also answered four items about the likelihood of the relationship ending (e.g., “She would consider ending the relationship”). These items were averaged (Study 2b α = 0.82). Measures were researcher-created for this study.

***Social Approval***

In Study 2b only, participants also answered six items regarding social approval of the relationship (e.g., “His family and friends would approve of his partner”). Items were coded so that higher scores indicated more social approval, then averaged (Study 2b α = 0.71). Measures were researcher-created for this study.

***Gender Nonconformity***

Participants used a 7-point agreement scale (1 = *strongly disagree*, 7 = *strongly agree*) to answer four items evaluating the target person’s feelings about their gender in their relationship (e.g., “She would feel like a ‘real woman’ in this relationship;” “He would not feel very “manly” in this relationship;” “She would feel like a stereotypical man in this relationship”). In Study 2b we accidentally worded the item, “He would not feel very “manly” in this relationship”, as “He would feel manly in this relationship.” We therefore reverse scored this item before averaging it with the other three items in the composite. Items were coded so that higher scores reflected greater gender nonconformity (e.g., feeling less feminine and/or more manly for the female target; feeling less masculine and/or more feminine for the male target) and then averaged (Study 2a α = 0.64; Study 2b α = 0.75). While the reliability of this scale is less-than ideal, the results were very similar when each item was analyzed individually, so we opted to use the index for simplicity. Measures were researcher-created for this study.

***Feelings of Inadequacy***

Participants used the same 7-point scale to answer seven items assessing the target’s feelings of inadequacy in their relationship (e.g., “She would feel inadequate in her relationship;” “He would worry whether his partner finds him attractive;” “She would worry about whether she is contributing enough to the relationship;” “He would feel good about himself in this relationship”). Items were coded so that higher scores indicated stronger feelings of inadequacy and then averaged (Study 2a α = 0.84; Study 2b α = 0.82). This measure was created for this study based on theories of relational insecurity (e.g., Stinson et al., 2016).

### Results

Table 2 reports the means, standard deviations, and correlations among measures for both studies. It is worth noting that participants’ perceptions of the target’s gender nonconformity and feelings of inadequacy were strongly correlated in both studies, consistent with our theorizing concerning gender threat.

**Table 2:** Descriptive Statistics and Correlations Among Study Variables for Participants in Study 2a and Study 2b

	Study 2a		Study 2b						
Variable	*M*	*SD*	*M*	*SD*	1	2	3	4	5
1. Relationship quality	5.56	0.96	5.73	0.89		− 0.58***	0.73***	− 0.63***	− 0.73***
2. Relationship dissolution			3.07	1.34			− 0.67***	0.47***	0.69***
3. Social approval			5.31	0.93				− 0.62***	− 0.67***
4. Gender nonconformity	2.88	1.13	2.67	1.11	− 0.51***				0.67***
5. Inadequacy	2.73	1.07	2.77	1.07	− 0.78***			0.52***	

*Note*. Correlations for Study 2a are below the diagonal and correlations for Study 2b are above the diagonal. Correlations are based on overall measures across experimental conditions

***p < .001

Preliminary analyses revealed that participant gender did not moderate the results we report for either study, so it was not included in the analyses we report. We explored sexual orientation as a moderator of the effects we report, and those results are presented in the OM (there were no effects in Study 2a and some inconsistent effects in Study 2b; https://osf.io/tuyj5/).

We used the same type of ANOVA in all our analyses for both studies, in which we entered relationship type (0 = MBR, 1 = FBR) and target (0 = Olivia, 1 = Zach) into a univariate analysis of variance (ANOVA) predicting each dependent variable. See Table 3 for results, which we will unpack in the following sections. For brevity, we will focus our interpretation on the significant interactions and main effects that are directly relevant to our theorizing; all main effects and non-significant tests are reported in Table 3.

**Table 3:** Dependent Measures in Study 2a (left) and Study 2b (right) as a Function of Relationship Type, Target Gender, and the Interaction Between Variables

	Study 2a					Study 2b				
Dependent variable	*df*	Mean square	*F*	*p*	η_*p*_^2^	*df*	Mean square	*F*	*p*	η_*p*_^2^
Relationship quality										
Relationship Type (MBR vs. FBR)	1	1.03	1.15	.285	0.001	1	4.46	5.66	.018	0.005
Target (Olivia vs. Zach)	1	8.41	9.31	.002	0.011	1	0.16	0.20	.651	0.000
Relationship Type X Target	1	3.57	3.95	.047	0.005	1	5.35	6.79	.009	0.006
Error	874	0.90				1217	0.79			
Relationship dissolution										
Relationship Type (MBR vs. FBR)						1	8.50	4.77	.029	0.004
Target (Olivia vs. Zach)						1	0.10	0.06	.810	0.000
Relationship Type X Target						1	0.35	0.20	.657	0.000
Error						1217	1.78			
Social approval										
Relationship Type (MBR vs. FBR)						1	28.50	33.50	< .001	0.027
Target (Olivia vs. Zach)						1	0.03	0.04	.841	0.000
Relationship Type X Target						1	0.11	0.13	.718	0.000
Error						1217	0.85			
Gender nonconformity										
Relationship Type (MBR vs. FBR)	1	177.20	209.29	< .001	0.193	1	40.42	34.14	< .001	0.027
Target (Olivia vs. Zach)	1	77.84	91.93	< .001	0.095	1	2.80	2.36	.124	0.002
Relationship Type X Target	1	120.36	142.15	< .001	0.140	1	31.32	26.45	< .001	0.021
Error	874	0.85				1216	1.18			
Feelings of inadequacy										
Relationship Type (MBR vs. FBR)	1	1.07	1.01	.314	0.001	1	2.55	2.37	.124	0.002
Target (Olivia vs. Zach)	1	14.88	14.09	< .001	0.016	1	1.64	1.52	.218	0.001
Relationship Type X Target	1	71.99	68.21	< .001	0.072	1	81.82	75.95	< .001	0.059
Error	874	1.06				1218	1.08			

*Note. FBR *female breadwinner relationship, *MBR *male breadwinner relationship

#### Relationship Quality

**Fig. 2:**
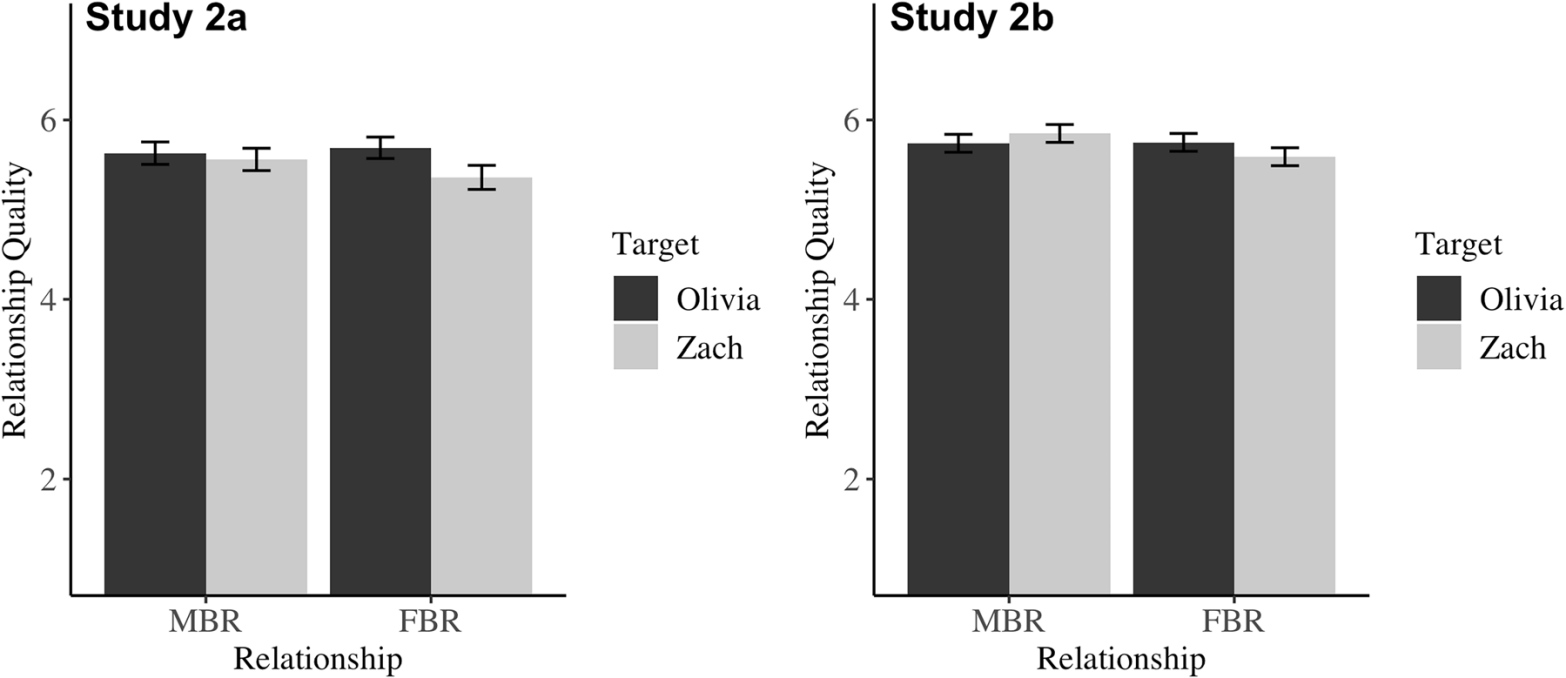
Mean perceived relationship quality as a function of relationship type and target in Study 2a (left) and Study 2b (right). Error bars reflect 95% confidence intervals

The anticipated interaction between relationship type and target was present in both studies. See Table 4; Fig. 2 for simple effects. Consistent with our hypotheses concerning precarious masculinity and heightened consequences for men who violate heterosexual scripts, participants in both studies perceived that Zach would have worse relationship quality in the FBR compared to the MBR, and that his relationship quality would be worse than Olivia’s in the FBR. However, Olivia’s perceived relationship quality did not differ between the FBR and the MBR; Olivia and Zach were perceived as being equally happy in the MBR.

**Table 4:** Simple Effects Within Target and Relationship Type in Study 2a (left) and Study 2b (right)

	Study 2a					Study 2b				
			95% CI					95% CI		
	Mean difference	Std. error	Lower bound	Upper bound	*η*_*p*_^*2*^	Mean difference	Std. error	Lower bound	Upper bound	*η*_*p*_^*2*^
Relationship quality										
Olivia: FBR (1) vs. MBR (0)	0.06	0.09	−0.120	0.238	0.000	0.01	0.07	−0.127	0.150	0.000
Zach: FBR (1) vs. MBR (0)	−0.20*	0.09	−0.374	−0.019	0.005	−0.25***	0.07	−0.398	−0.109	0.010
MBR: Zach (1) vs. Olivia (0)	−0.07	0.09	−0.250	0.113	0.001	0.11	0.07	0.017	0.295	0.002
FBR: Zach (1) vs. Olivia (0)	−0.32***	0.09	−0.499	−0.149	0.015	−0.16*	0.07	−0.295	−0.017	0.028
Gender nonconformity										
Olivia: FBR (1) vs. MBR (0)	0.16	0.09	−0.015	0.332	0.004	0.04	0.09	−0.126	0.213	0.000
Zach: FBR (1) vs. MBR (0)	1.64***	0.09	1.469	1.813	0.287	0.69***	0.09	0.508	0.862	0.045
MBR: Zach (1) vs. Olivia (0)	0.15	0.09	−0.031	0.321	0.003	−0.23*	0.09	−0.401	−0.049	0.005
FBR: Zach (1) vs. Olivia (0)	1.34***	0.09	1.168	1.507	0.216	0.42***	0.09	0.246	0.587	0.019
Feelings of inadequacy										
Olivia: FBR (1) vs. MBR (0)	−0.50***	0.10	−0.697	−0.310	0.029	−0.43***	0.08	−0.588	−0.265	0.022
Zach: FBR (1) vs. MBR (0)	0.64***	0.10	0.451	0.835	0.047	0.61***	0.09	0.441	0.779	0.040
MBR: Zach (1) vs. Olivia (0)	−0.31**	0.10	−0.509	−0.117	0.011	−0.45***	0.09	−0.612	−0.278	0.022
FBR: Zach (1) vs. Olivia (0)	0.83***	0.10	0.645	1.023	0.079	0.59***	0.08	0.429	0.754	0.040

*Note. FBR *female breadwinner relationship, *MBR *male breadwinner relationship

*p < .05; **p < .01; ***p < .001

***Relationship Dissolution***

Participants in Study 2b perceived that the FBR was more likely to end than the MBR (*Ms* = 3.14 and 2.98, *SDs* = 1.35 and 1.32).

***Social Approval***

Participants in Study 2b also perceived that FBRs would garner less social approval than MBRs (*Ms* = 5.16 and 5.46, *SDs* = 0.95 and 0.89).

#### Gender Threat

***Gender Nonconformity***

**Fig. 3:**
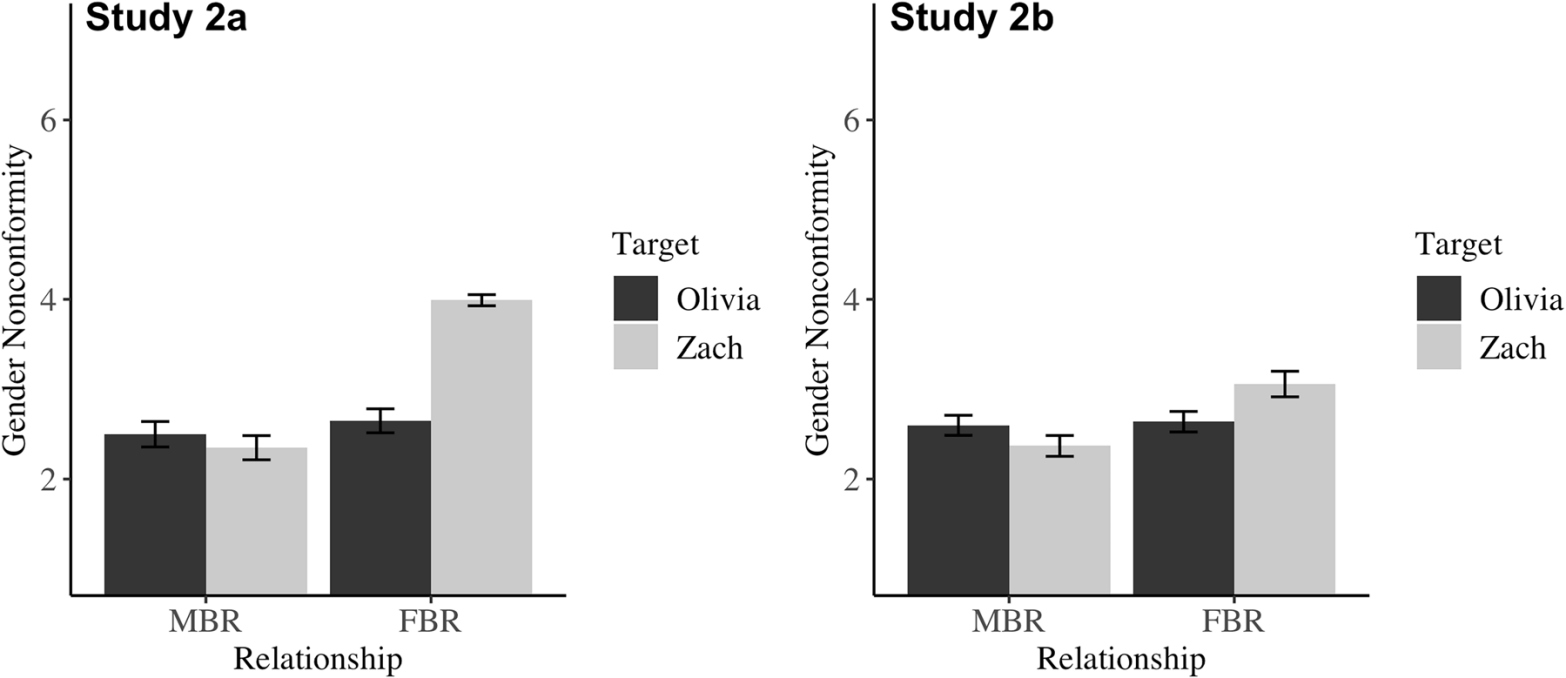
Mean perceived gender nonconformity as a function of relationship type and target in Study 2a (left) and Study 2b (right). Error bars reflect 95% confidence intervals

Both studies showed the anticipated interaction between variables. See Table 4; Fig. 3 for simple effects decomposing this interaction. Consistent with our hypotheses, participants perceived that Zach would feel more gender nonconformity in the FBR than the MBR. As expected, they also thought that Zach’s feelings of gender nonconformity would be stronger than Olivia’s feelings of gender nonconformity within the FBR. However, as with relationship quality, there were no differences in perceived gender nonconformity for Olivia across relationship types (though participants in Study 2b did perceive that Olivia would experience slightly more gender nonconformity than Zach in an MBR, which was unexpected).

***Feelings of Inadequacy***

**Fig. 4:**
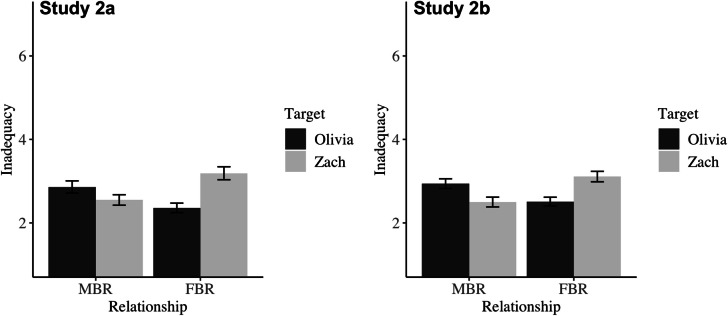
Mean perceived inadequacy as a function of relationship type and target in Study 2a (left) and Study 2b (right). Error bars reflect 95% confidence intervals

Both studies showed the anticipated interaction between relationship type and target. See Table 3; Fig. 4 for simple effects. Consistent with our hypotheses, participants in both studies perceived that Zach would experience greater feelings of inadequacy in an FBR than in an MBR, and he was perceived to experience more inadequacy than Olivia in the FBR. Unexpectedly, however, participants perceived that Olivia would experience less inadequacy in the FBR than the MBR, and participants thought that she would experience more inadequacy than Zach in the MBR. The overall pattern of results suggests that observers equated dependence with inadequacy and breadwinning with self-worth, for both men and women.

### Discussion

The results of these experiments further corroborated the qualitative themes of Study 1 and confirmed our overarching hypotheses by revealing people’s implicit knowledge of heterosexual scripts, and the consequences for violating those scripts. Consistent with Study 1, people believed that FBRs were poorer quality relationships that were more susceptible to divorce compared to MBRs. FBRs were also expected to garner less social approval than their more script-conforming counterparts. Moreover, consistent with theories of precarious masculinity (e.g., Vandello et al., 2008), men in FBRs were believed to suffer to a greater extent than women in these relationships. Specifically, participants expected that the man in an FBR would experience worse relationship quality, greater feelings of gender nonconformity, and greater feelings of inadequacy than his breadwinning female partner. In contrast, participants did not think breadwinning status would affect the female partner’s outcomes in the narrative they evaluated. Thus, if we take MBRs as the comparison standard, there appears to be a downward effect for perceptions of men in FBRs and a mostly null effect for perceptions of women’s experiences in FBRs. The pattern of observed null results for Olivia suggests that heterosexual scripts may be shifting for some women in some contexts, which is consistent with recent research suggesting that female gender stereotypes have become more agentic in recent years (Eagly et al., 2020). However, the overall consistency between portrayals of FBRs as gender troublemakers in the media accounts in Study 1 and in participants’ evaluations in the current experiments suggests that heterosexual scripts, and the breadwinning sub-script, informed the results of both studies.

## Study 3: Relationship Experiences in FBRs

In our final study, we tested our hypotheses concerning the relations between breadwinner status and gender nonconformity, feelings of inadequacy, and relationship quality among a sample of more than 500 married adults in heterosexual relationships. We predicted that compared to people in MBRs, people in FBRs would report worse relationship quality, and we used serial mediation analyses to examine whether gender nonconformity and inadequacy (i.e., gender threat) explained, in part, why FBRs reported worse relationship quality than their MBR peers. Further, we expected that the indirect link between breadwinning status and relationship quality would be stronger for men than for women because men experience greater gender threat than women in FBRs due to precarious masculinity.

### Method

This study was conducted as part of a broader honors thesis project about work and family life during COVID-19 in 2020. Additional measures, supplemental analyses, data, and analysis code are available in the OM on the OSF (https://osf.io/tuyj5/). Additional data from participants in Study 3, completely unrelated to the hypotheses or results reported here (i.e., reports of whether they were friends with their spouse before they became romantically involved), were reported in Stinson et al. (2022). This research received approval from the Human Research Ethics Office at the University of Victoria, Canada (approval number: 20–0491).

#### Participants

A priori power analyses using G*Power (Faul et al., 2007) indicated that to detect a population *f*^2^ of 0.02 (a small effect) with 80% power and α = 0.05, we would need a sample of 550 people. Thus, we aimed to recruit 750 participants to account for exclusions and to ensure that our sample included enough parents to meet the needs of the broader thesis project. Participants were recruited through MTurk and needed to be married adults (age 18+) living in the United States. They were paid $2.50 in appreciation for their time.

The original sample size was 750, but 55 participants (7%) were excluded for the following reasons: completing the survey in under 2 min (*n* = 14), duplicate IP addresses (*n* = 2), or failing one or more of the validation questions (e.g., “for validation purposes, select E as a response;” *n* = 39). Two participants were also excluded who did not report their or their partner’s gender or reported a nonbinary gender, and 35 were excluded because they were in same-gender/sex relationships. We excluded these participants because our hypotheses were based on heterosexual scripts, and LGBTQ + people typically do not ascribe to those scripts (e.g., Rose, 2000). We present some supplemental analyses for LGBTQ + participants in the OM (https://osf.io/tuyj5/). Unfortunately, data for another 145 participants (19%) had to be excluded due to a coding error for survey responses indicating own or partner income greater than $7000 per month. Thus, all participants had a combined household income less than or equal to $14,000 per month (see the OM [https://osf.io/tuyj5/] for more details about this exclusion). This exclusion resolved the coding error and had the desirable side-effects of yielding a normal distribution of income scores and bringing the average household income in our sample in line with the median U.S. household income for 2020 (i.e., around $71000 for our sample versus about $67,000 per the census; United States Census Bureau, 2024) The full sample had neither of these characteristics.

These exclusions resulted in a final sample of 511 participants in heterosexual relationships (*M*_age_ = 37.8 years, *SD*_age_ = 8.2 years; 56.4% women [*n* = 288], 43.6% men [*n =* 223]. Participants’ ethnic identities were as follows: *n =* 42 (8.2%) Black/African American, *n =* 24 (4.7%) East Asian, *n =* 7 (1.4%) East Indian or South Asian, *n =* 19 (3.7%) Latino/Latina/Latinx, *n* = 9 (1.8%) Native American/Native Hawaiian/other Pacific Islander, and *n* = 424 (83%) White/Caucasian). Sensitivity analyses using G*Power (Faul et al., 2007) indicated that with this sample size, 80% power, and α = 0.05, we could detect a population *f*^2^ of 0.025.

#### Procedure

Participants on MTurk who met the inclusion criteria saw an invitation to take a survey on changes in work and family life during COVID-19. Interested participants were directed to Qualtrics where they first read the implied consent and then proceeded through the survey questions and lastly read a debriefing letter concerning our research goals.

#### Measures

***Income***

Participants reported their own and their partner’s approximate current monthly income, including public assistance from other sources, using 8-point scales (0 – *not applicable/not earning*, 1 – *income less than $1000*, 4 – *income between $3000 and $4000*, 7 – *income between $6000 and $7000*). We also summed participant and partner income to yield a *total couple income* score, and then divided the income earned by the female partner by the total couple income to obtain the *female partner’s proportion of income* (POI).

***Relationship Quality***

Participants used a 7-point scale (1 – *strongly disagree*, 7 – *strongly agree*) to respond to a series of questions about their relationship outcomes, adapted from similar measures members of our lab used in previously-published research (Fisher et al., 2021) and based on theory and research concerning attachment security (Cameron et al., 2012), satisfaction and commitment (Rusbult, 1980), and perceived regard (Gaucher et al., 2012).

**Overall Relationship Quality.** Participants indicated their trust (“I trust my spouse or common-law partner”), security (“I feel secure in my relationship with my spouse or common-law partner”), satisfaction (“I feel satisfied in my relationship with my spouse or common-law partner.”), and commitment (“I am committed to maintaining this relationship.”) in their relationship, which were averaged to form a reliable measure of *overall relationship quality* (α = 0.88).

**Attraction to Spouse.** They also indicated their *attraction to their spouse* with two items (“I am attracted to my spouse or common-law partner;” “I feel sexually attracted to my spouse or common-law partner”), which were averaged (α = 0.89).

**Mutual Admiration.** Participants also responded to five items tapping their feelings of *mutual admiration* in their relationship (e.g., “I feel attractive to my spouse or common-law partner;” “I respect and appreciate my spouse or common-law partner;” “I feel proud of my spouse or common-law partner”), which were averaged (α = 0.89).

***Gender Nonconformity***

Participants again used a 7-point scale (1 – *strongly disagree*, 7 – *strongly agree*) to report their feelings of *femininity* (“I feel feminine or womanly in this relationship;” “I feel more like the “woman” in this relationship;” averaged, α = 0.94) and *masculinity* (“I feel masculine or manly in this relationship;” “I feel more like the “man” in this relationship;” averaged, α = 0.95) in their relationship. This measure was created for this study based on measures used in Study 2. We created a variable called *gender nonconformity* that comprised feelings of femininity/womanliness for men and feelings of masculinity/manliness for women.

***Feelings of Inadequacy***

Participants used the usual 7-point scale to answer five items tapping their feelings of inadequacy in their relationship (e.g., “I feel inadequate in my relationship;” “I worry whether my partner finds me attractive;” “I worry about whether I am contributing enough to the relationship”), which were averaged (α = 0.88). This measure was created for this study based on theories of relational insecurity (e.g., Stinson et al., 2016).

### Results

#### Preliminary Analyses

Preliminary analyses indicated that the three measures of relationship quality – overall relationship quality, attraction to spouse, and mutual admiration – were very highly correlated with one another (*r*s = 0.82 to 0.90, all *ps* < 0.001), loaded onto a single factor in an exploratory principle components factor analysis, and had similar associations with other variables, so they were averaged to yield a reliable *relationship quality* index (α = 0.93). Further, couple total monthly income (*M* = 6.93 [$5930], *SD* = 2.97 [$2970]) did not moderate any of the results we describe below.

As shown in Table 5, and as expected, as the proportion of income earned by the female partner in the participants’ marriage was higher, relationship quality was lower and feelings of gender nonconformity and inadequacy were higher. The zero order correlations among other variables were also all in the expected directions, though the correlation between gender nonconformity and inadequacy was surprisingly strong. This suggests that most people in this sample were uncomfortable with gender nonconformity.

**Table 5:** Descriptive Statistics and Correlations Among Study Variables for Participants in Heterosexual Relationships in Study 3

Variable	*M*	*SD*	2	3	4
1. Female partner’s POI	0.41	0.21	− 0.13**	0.17***	0.11*
2. Relationship quality	5.90	1.07		− 0.26***	− 0.40***
3. Gender nonconformity	2.66	1.90		-	0.74***
4. Inadequacy	3.07	1.57		-	-

*Note. POI *proportion of household income earned

*p < .05; **p < .01; ***p < .001

#### Main Analysis

**Fig. 5:**
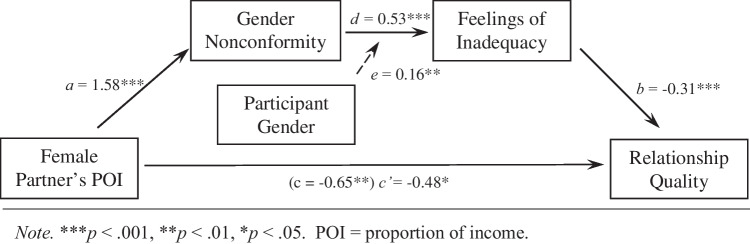
Moderated Serial-Mediation Model for Participants in Heterosexual Relationships in Study 3

We examined whether gender threat (i.e., gender nonconformity linked to feelings of inadequacy) explained in part the observed negative association between breadwinner status and relationship quality, and whether this indirect path linking breadwinning status to relationship quality was stronger for men than for women. These hypotheses correspond to a serial moderated mediation model, which is depicted along with the results of our analyses testing the model in Fig. 5. We used Hayes’ (2022) PROCESS Macro version 4.0 in SPSS to test our moderated serial mediation model, using 10,000 bootstrap samples, whereby X = Female POI, Y = Relationship Quality, M_1_ = Gender Nonconformity, M_2_ = Inadequacy, and W = Participant Gender/Sex. All predictor variables were mean-centered in this analysis. We initially tested Model 92 whereby W moderated all paths in the model and then pared the model by eliminating non-significant moderated paths, following Hayes’ (2022) recommendations. The final model (Model 91 in PROCESS) is described in Table 6 and depicted in Fig. 5 (full PROCESS results are reported in the OM; https://osf.io/tuyj5/). In sum, the model accounted for about 17% of the variance in participants’ reported relationship quality, *R*^2^ = 0.17, *F*(3, 504) = 34.77, *p* < .001.

**Table 6:** PROCESS Results Testing Moderated Serial-Mediation Model for Participants in Heterosexual Relationships in Study 3

**Regression analyses**						
Step in Analysis	*β*	*b* [CI]	*SE*	*t*	*p*	*R*^*2*^
Outcome Variable: Gender Nonconformity						0.03
Constant		0.01 [−0.15, 0.18]	0.08	0.18	.859	
Female Partner’s POI	0.17	1.58 [ 0.79, 2.36]	0.40	3.97	< .001	
Outcome Variable: Inadequacy						0.55
Constant		−0.01 [−0.14, 0.11]	0.06	−0.24	.807	
Female Partner’s POI	− 0.02	−0.16 [−0.62, 0.30]	0.23	−0.68	.498	
Gender Nonconformity	0.64	0.53 [ 0.46, 0.60]	0.04	15.18	< .001	
Participant Gender	0.01	0.02 [−0.17, 0.21]	0.10	0.22	.825	
Gender Nonconformity x Participant Gender	0.14	0.16 [ 0.06, 0.26]	0.05	3.27	.001	
Outcome Variable: Relationship Quality						0.17
Constant		5.89 [ 5.81, 5.98]	0.04	135.10	< .001	
Female Partner’s POI	− 0.09	−0.48 [−0.90, 0.07]	0.21	−2.27	.024	
Gender Nonconformity	0.09	0.05 [−0.02, 0.12]	0.03	1.45	.150	
Inadequacy	− 0.46	−0.31 [−0.39, −0.23]	0.04	−7.61	< .001	
**Indices of mediation**						
Test		*Effect [CI]*	*SE*	*t*	*p*	
Total Effect of X on Y		−0.65 [−1.09, −0.20]	0.23	−2.87	.004	
Direct Effect of X on Y		−0.48 [−0.90, 0.07]	0.21	−2.27	.024	
Conditional Indirect Effect of X on Y						
Women		−0.26 [−0.42, −0.13]	0.07			
Men		−0.34 [−0.53, −0.18]	0.09			
Index of Moderated Mediation		−0.08 [−0.15, 0.03]	0.03			

*Note*. Total effect of X on Y and standardized coefficients (*β*) for all tests were derived from linear regressions testing the same model reported above, as PROCESS does not produce those statistics

*POI *proportion of income, *X* Female Partners’ POI, *Y *Relationship Quality

As we have already reported (see Table 5), increases in income earned by the female partner in the participants’ marriage predicted heightened feelings of gender nonconformity for everyone; this path was not moderated by gender (*path a*). In turn, consistent with our theorizing concerning the nature of gender threat, gender nonconformity predicted feelings of inadequacy for everyone (path *d*), but consistent with precarious manhood theory, the association was stronger for men, *b* = 0.69 [0.62, 0.76], *SE* = 0.04, than for women *b* = 0.53 [0.46, 0.60], *SE* = 0.04 (path *e*). In turn, heightened feelings of inadequacy predicted worse relationship quality for everyone (path *b*). Finally, the indirect path in the model (i.e., *a x d x b*) was also present and moderated by gender, as evidenced by the significant index of moderated mediation (see Table 6). As predicted, gender threat explained in part why participants in FBRs experienced worse relationship quality than their MBR counterparts; gender threat also explained a greater proportion of the association between breadwinner status and relationship quality for men than for women. None of the other possible indirect paths in the model were statistically significant (i.e., Female Partner’s POI $\rightarrow$ Gender Nonconformity $\rightarrow$ Relationship Quality; Female Partner’s POI $\rightarrow$ Feelings of Inadequacy $\rightarrow$ Relationship Quality).

### Discussion

The results of our third study offer novel insight into why FBRs seem to struggle compared to their MBR counterparts. Consistent with previous research (e.g., Blom & Hewitt, 2020), compared to their MBR counterparts, men and women in FBRs experienced worse relationship quality. However, we found evidence of a novel explanatory mechanism behind these poor outcomes: gender threat. Men and women in FBRs reported heightened feelings of gender nonconformity and feelings of inadequacy (i.e., gender threat) compared to their counterparts in MBRs, and as in Studies 1 and 2a/2b, gender threat was experienced more strongly by men than by women. In turn, gender threat explained FBRs worse relationship quality. Thus, men’s greater susceptibility to gender threat may explain why they seem to fare worse than women in FBRs.

## General Discussion

Using a combination of qualitative and quantitative methods, the current research investigated the content of heterosexual scripts and the punishing social consequences for female breadwinner couples who violate those scripts. Across studies, our results converged to reveal a stigmatizing portrayal of FBRs as less desirable, worse quality, and less stable than MBRs. Our research also identified a novel explanatory mechanism for FBRs’ poor relationship outcomes, namely, gender threat (i.e., co-occurring feelings of gender nonconformity and inadequacy). Being in a FBR was expected to (and actually did) elicit greater feelings of gender threat, particularly among men in those relationships, and in turn, heightened feelings of gender threat predicted worse relationship quality. Across all the studies we reported, gender threat and its deleterious effect on relationship quality was worse for men than women in FBRs. As perplexing as such results may seem to characters from the future feminist utopia that is Star Trek (as we imagined at the opening of this manuscript), the social, interpersonal, and intrapersonal consequences of violating heterosexual scripts are all too real for the women and men participating in FBRs today, and they are testament to the pervasiveness and regulatory power of heterosexual scripts in Western culture.

That men are expected to (and do) suffer to a greater extent than women in FBRs is consistent with theories of precarious manhood and cultural androcentrism. Theories of precarious manhood propose that to prove their masculinity, men must avoid appearing feminine (e.g., Vandello et al., 2008; see also Vandello & Bosson, 2013, for a review). Our findings suggest that this aversion to appearing feminine is at the core of men’s poor outcomes in FBRs, because it was men’s feelings of femininity, specifically, that predicted their feelings of inadequacy and in turn, their worse relationship outcomes. Indeed, our supplemental analyses confirmed that low feelings of masculinity are not substitutable for high feelings of femininity in the mediation analysis for men in Study 3. This suggests that efforts to bolster men’s sense of masculinity in FBRs, including the advice in Study 1 for women to ‘forget’ their wallet at home so their male partner can pay, may not be effective at combatting gender threat.

Consistent with theories of cultural androcentrism (Bailey et al., 2019), reactions to the script violations posed by FBRs seemed to center men’s suffering. Indeed, prior research finds that men and women demonstrate a particular sensitivity for protecting men’s masculinity from perceived threats (Vandello et al., 2008), and women are explicitly socialized to protect and bolster men’s egos (Impett & Peplau, 2003). Thus, heightened concern for men’s well-being in FBRs may serve the more nefarious purpose of legitimizing male dominance in heterosexual relationships (Jost & Hunyady, 2003). It may also help to explain why heterosexual scripts, which prioritize male dominance and supremacy, have remained largely unchanged for decades (Cameron & Curry, 2020).

Women’s career advancement, higher self-esteem, and heightened well-being in FBRs was also framed as occurring at the expense of men’s well-being – either explicitly in Study 1 or implicitly because of co-occurrence in Studies 2a/2b and 3 – which suggests that economic gains for women in the context of heterosexual relationships may be perceived as losses for men in those same relationships. This kind of zero-sum thinking is a well-documented impediment to gender equality in the workplace and in society more broadly (Ruthig et al., 2017; Zehnter et al., 2021), and is characterized by an exaggerated concern that women’s progress will ultimately undermine men’s longstanding privileges. This type of zero-sum thinking is also based on the tacit association of relational and societal benefits with the breadwinning role (e.g., decision-making power within the relationship, access to resources outside the relationship) and relational and societal drawbacks with the caregiving role (e.g., relational dependency, lower societal power; Bear & Glick, 2017), alongside the motivation to preserve men’s greater access to these breadwinning benefits.

Interestingly, however, when it comes to their actual experiences, breadwinner women also experienced heightened feelings of gender nonconformity and inadequacy. Although womanhood is generally perceived as stable (e.g., Vandello et al., 2008), these findings are testament to the power of the heterosexual script and the notion that perceiving oneself as gender nonconforming can also undermine women’s well-being. This is consistent with past qualitative research which finds that breadwinning women experience feelings of worry and guilt about their role (Meisenbach, 2010). Of course, there may also be other mechanisms at play. Female breadwinners tend to perform more housework than their counterparts in more economically-egalitarian relationships (Bittman et al., 2003), perhaps in an effort to conceal or compensate for their perceived script violations. Female breadwinners may feel that they have emotional, organizational, and physical responsibilities at home on top of their responsibilities as primary earners outside of the home, the strain of which may be particularly pronounced when their husbands and partners are also experiencing heightened gender threat and are therefore not doing their part to share the communal and caregiving responsibilities within the relationship (Eisler & Skidmore, 1987; Gillespie & Eisler, 1992). Ultimately, given that the number of female breadwinner households is only expected to increase, it is more pertinent than ever to subvert heterosexual scripts and improve experiences within FBRs. We discuss how this may be accomplished in the following sections.

### Limitations and Future Research Directions

While our research offers a rich and nuanced account of the content of heterosexual scripts and reactions to FBRs who violate those scripts, it also has limitations. For instance, Study 1 sampled newspaper and magazine articles from just three Western countries (Canada, the US, and the UK), and it is unclear whether we would observe similar results from other countries and cultures. Furthermore, our sample of articles were from before the COVID-19 pandemic and data for Study 3 were collected during the pandemic, which wrought widespread social change including dramatic shifts in gendered labour (e.g. Fisher & Ryan, 2021; Fisher et al., 2024). Perhaps those changes also altered heterosexual scripts and/or reactions to relationships like FBRs that violate those scripts in Study 1. The pandemic also may have moderated feelings of gender threat among the FBRs in Study 3. A comparison of changes in media representations of heterosexual scripts and FBRs pre-to-post pandemic would be fascinating. Replicating Study 3 post-pandemic would also be edifying. We suspect that, if anything, the pandemic heightened media and personal sensitivity to the threat posed by gender troublemakers like FBRs. Indeed, the anti-feminist and anti-trans social and political movements happening in all the countries from which we sampled articles suggests that reactivity to gender troublemaking was indeed exacerbated by the stress and uncertainty of the pandemic.

Study 2a and 2b involved a hypothetical scenario whereby a woman received a raise and became the breadwinner in her relationship. This scenario reflects just one of the many ways that a relationship can become an FBR. Some research suggests that relationships that transition into FBRs, rather than those that begin as FBRs, are more likely to struggle (Foster & Stratton, 2020; Syrda, 2020). Moreover, because men still earn more money than women in Western countries and globally (World Economic Forum, 2023), it is still relatively rare for a woman to out-earn her male partner when both are employed and working full-time. It is more common for heterosexual relationships to become FBRs when the male partner loses his job (Drago et al., 2004). Thus, FBRs may be associated with worse outcomes, in part, because they experience a higher degree of financial instability than MBRs (Glynn, 2019). Nonetheless, our hypothetical scenario offers a conservative test of whether something as minor as a raise for the female partner (without an explicit loss for the male partner) would be enough to upset the heterosexual script. That we were able to triangulate our findings across multiple methods and mediums lends confidence to the robustness of these results. However, future research should test to what extent the scripted expectations observed in our research extend to other scenarios. Because heterosexual scripts are so heavily gendered, we expect that people may make similar attributions (albeit perhaps stronger or weaker), especially about men’s gender nonconformity and well-being, regardless of how he finds himself in a relationship with a higher-earning woman.

Moreover, our studies did not examine the effects of class and race/ethnicity, but these factors are likely to affect the breadwinner script. For example, in the US, Black mothers are more likely to be breadwinners than White mothers, and have a longer, more consistent history of working outside the home (Glynn, 2019). Female breadwinner relationships are therefore more normative in Black communities, and potentially less detrimental to well-being and relationship quality as a result. The effects of class and race/ethnicity are difficult to tease apart in the current research given our samples were predominately White and middle-class. Future research should examine to what extent these scripts operate across class and race/ethnicity, taking into account how the social context and histories of different groups may shape the scripts.

Finally, another important avenue for future research is to explore how these scripts are perceived and experienced in LGBTQIA + communities. Queer people are “dually socialized” in that they are exposed to both heteronormativity and pressures to subvert and deviate from heterosexual norms (Green, 2010, p. 401; Lamont, 2017). Nonetheless, queer communities have a long history of resistance to heterosexual norms and may therefore be better positioned to critique and dismantle heteronormative scripts when they arise in their relationships (Lamont, 2017). For this reason, we wonder whether couples who more easily adapt to being in an FBR include one or more queer people who are more practiced in gender troublemaking. After all, queer couples often write intrapersonal scripts that reject heterosexual norms, like negotiating more egalitarian divisions of labor in their relationships that are based on attributes like personal preferences and time availability, rather than gender role expectations (Kelly & Hauck, 2015). Likewise, whereas some couples, queer or straight, may find adhering to heterosexual scripts comforting and gender-affirming, others may find them constraining and at odds with their authentic gender expression. More research is needed to investigate how queer people navigate their relationships in relation to dominant norms and narratives (Lamont, 2017).

### Practice Implications

Our findings may be informative for policymakers, organizations, practitioners, and people in romantic relationships. Policymakers should be aware that propaganda about the ‘dangers’ of entering an FBR may undermine women’s career advancement and progress toward gender equality more broadly. For example, women may worry that becoming the breadwinner in their relationship will undermine their own and their male partners’ well-being. These concerns may lead women to hold back in their careers or to pass up opportunities that would otherwise allow them to get ahead. At the same time, men’s fear of being feminized may lead them to under-appreciate the benefits of having a higher earning partner. Policymakers should consider whether campaigns that counter the stigmatizing portrayals of FBRs may be needed to counteract the effects of the negative media portrayals that we observed in Study 1.

This research also has implications for workplaces and organizations. Longstanding gender inequalities in organizational contexts (e.g., gender pay gap, gendered hierarchies) reinforce the male breadwinner model and heterosexual scripts. Heterosexual scripts in close relationships can also permeate into the workplace. For example, the structure of men’s marriages, specifically whether they have employed or unemployed spouses, can influence their attitudes toward female coworkers and gender equality in the workplace (Desai et al., 2014; Mikołajczak et al., in press). Thus, organizations can and must do their part to disrupt heterosexual scripts. One way that organizations can begin to do so is through the implementation of workplace policies and practices that encourage equal sharing of caregiving responsibilities between women and men. Normalizing men’s uptake of childcare and parental leave may help to untether masculinity from the primary provider role and encourage the expansion of masculinity to include communal traits and caregiving responsibilities (Croft et al., 2015; Reid, 2018; Tinsley et al., 2015). Organizations may also be well positioned to challenge heterosexual scripts by supporting the career advancement of women alongside efforts to normalize men’s engagement in supportive, caregiving roles within the workplace and the home.

Therapists and practitioners working with female-breadwinner couples should also be aware that the distress that FBRs experience may stem from social stigma and gender threat. Indeed, our results suggest that heterosexual couples internalize and judge themselves according to scripted expectations. Thus, those who most strongly endorse the male breadwinner script are likely to be at greatest risk of poor well-being in FBRs (Gonalons-Pons & Gangl, 2021). In contrast, couples who are aware of this script may be better positioned to dismantle it and re-imagine new alternatives in their relationships. The results of Study 1 suggest that couples who reject heterosexual scripts in favor of egalitarian interpersonal scripts may enjoy better relationship quality than their MBR counterparts who embody those scripts, and they certainly fare better than people in FBRs who internalize social stigma and experience gender nonconformity as a threat to their worth as a partner and as a woman or man.

Likewise, reducing the social stigma surrounding FBRs (and the internalization of such stigma) could also help to eliminate the negative self-judgement (i.e., inadequacy) that is necessary for gender threat to occur. Both of these potential inoculations against gender threat within FBRs could be facilitated by engaging with feminist and queer community and theorizing which often centers on disrupting heterosexual scripts through community-engaged gender troublemaking (e.g., Ahmed, 2006; Lamont, 2017). Practitioners could work with FBR couples, and couples more generally, to interrogate the scripted beliefs they hold and to develop more idiosyncratic and egalitarian norms within their relationships and more flexible and inclusive gender identities. Practitioners should also work to uncover their own biases about FBRs, which may implicitly influence the assumptions they make about the relational dynamics of such couples and the care they offer such clients. They should be especially careful to avoid the types of coercive efforts we saw in Study 1, aimed at encouraging breadwinning women to conform to heterosexual norms as a means of reducing male suffering.

It is also important to be aware that the fear of feminization that some men experience in FBRs may also lead them to lash out or engage in compensatory behaviors to re-establish their masculinity (Morgenroth & Ryan, 2021; Stanaland et al., 2023). Past research suggests that men are more likely to cheat when their female partners earn more than them (Munsch, 2018). Other research shows that men behave more assertively toward female superiors when their masculinity is threatened (Netchaeva et al., 2015). Given the theoretical link between threats to men’s masculinity and aggression (Bosson & Vandello, 2011), practitioners should be aware that intimate partner violence may also be a greater risk in these relationships.

### Conclusion

Prior research has found that couples who violate the breadwinner script are stigmatized and expected to struggle (e.g., Brescoll & Uhlmann, 2005; Pierce et al., 2013; Syrda, 2020). Our research goes one step further by demonstrating a novel mechanism behind the stigma and suffering: gender threat, comprising feelings of gender nonconformity and inadequacy. By examining how gender threat affects couples who challenge the breadwinner script, our study reveals how outdated notions about gender roles can prevent modern female breadwinner couples from enjoying the same intrapersonal, interpersonal, and social well-being as their more script-abiding counterparts. In this way, heterosexual scripts can create barriers not only to gender equality in close relationships but also in the broader context of economic gender equality. Therefore, our research supports the conclusion that it is not female breadwinner relationships themselves that are inherently “dangerous,” but rather the cultural scripts surrounding them.

## Supplementary Information

Below is the link to the electronic supplementary material.

ESM 1 (DOCX 100 KB)

## Data Availability

Participant data is available upon request from the corresponding author.

## Code Availability

The materials and analytic code for this study are available at https://osf.io/tuyj5/.
