# Supplementary material for: “A Recipe for Disaster?”: Female-Breadwinner Relationships Threaten Heterosexual Scripts
Source: Sex Roles. 2025 Feb 19;91(3):16. doi: 10.1007/s11199-025-01560-y (PMC11839713; doi:10.1007/s11199-025-01560-y)
Supplement: Supplementary file 1 — (DOCX 100 KB) [file 11199_2025_1560_MOESM1_ESM.docx]

Online Materials for

“A Recipe for Disaster?”:

Female-Breadwinner Relationships Threaten Heterosexual Scripts

Published in *Sex Roles: A Journal of Research*

by

Alexandra N. Fisher

Danu Anthony Stinson

Anastasija Kalajdzic

Hannah E. Dupuis

Erin E. Lowey

Elysia Desgrosseilliers

Annie MacIntosh

Table of Contents

Study 1 3

Study 2a 17

Study 2b 18

[Study 3 19](#_Toc712442070)

# **Study 1**

| **Table S1**  *Female Breadwinner Article Descriptives* | | | | | | | | |
| --- | --- | --- | --- | --- | --- | --- | --- | --- |
|  | Article Title | Search Engine | Name of Article Source | Country of Source | Type of Article | Date Published | Political Leaning of Source | Author Name |
| 1 | [7 Tips for Breadwinner Wives Feeling the Strain](https://www.forbes.com/sites/nextavenue/2013/06/05/7-tips-for-breadwinner-wives-feeling-the-strain/#2d6f37bc3d8b) | Google | Forbes | USA | Magazine | 05-Jun-13 | Conservative | Kerry Hannon |
| 2 | [A Brief History of Dumb Things Men Have Said](https://prospect.org/article/brief-history-dumb-things-men-have-said) | Google | The American Prospect | USA | Magazine | 04-Jun-13 | Liberal | E. J. Graff |
| 3 | [Ali Wong Nails the Downside of Being a Woman Breadwinner](https://www.refinery29.com/en-us/2018/05/199107/ali-wong-netflix-hard-knock-wife) | Google | Refinery 29 | USA | News | 14-May-18 | N/A | Maia Efrem |
| 4 | [An American Role-Reversal: Women the New Breadwinners](https://www.usatoday.com/story/news/nation/2013/03/24/female-breadwinners/2015559/) | Google | USA Today | USA | Newspaper (Factual) | 24-Mar-13 | No bias | Dennis Cauchon |
| 5 | [Are Female Breadwinners a Recipe for Disaster?](https://nypost.com/2014/04/30/are-female-breadwinners-a-recipe-for-disaster/) | Google | NY Post | USA | Newspaper (Opinion) | 30-Apr-14 | Conservative | Sara Stewart |
| 6 | [As 40 Percent of Women Now Out-Earn Spouses, Black Women Cope Well in New Age of 'Breadwinner Moms'](https://thegrio.com/2013/06/14/african-american-women-coping-well-in-new-age-of-breadwinner-moms-in-which-40-percent-of-women-out-earn-spouses/) | Google | The Grio | USA | News | 14-Jun-13 | N/A | N/A |
| 7 | [Back to the '50s? Many Teens Say Man Should Be in Charge at Home](https://www.livescience.com/58481-teens-want-man-in-charge-at-home.html) | Google | Live Science | USA | News | 31-Mar-17 | N/A | Stephanie Pappas |
| 8 | [Being the Breadwinner: A Blessing and a Curse](https://www.marketwatch.com/story/being-the-breadwinner-a-blessing-and-a-curse-2014-09-19) | Google | Market Watch | USA | News | 20-Sep-14 | N/A | Mackenzie Dawson |
| 9 | [Breadwinner Moms](https://www.pewsocialtrends.org/2013/05/29/breadwinner-moms/) | Google | Pew Research Centre | USA | News/ Statistics | 29-May-13 | N/A | Wendy Wang Kim Parker Paul Taylor |
| 10 | [5 Signs that Attitudes Towards Female Breadwinners are Changing](https://www.marieclaire.co.uk/life/work/changing-society-s-attitude-towards-the-female-breadwinner-75439) | Google | Marie Claire | England | Magazine | 08-Apr-16 | N/A | N/A |
| 11 | [Black Women: Supporting Their Families—With Few Resources](https://www.theatlantic.com/business/archive/2017/06/black-women-economy/530022/) | Google | The Atlantic | USA | Magazine | 12-Jun-17 | Leans left | Gillian B. White |
| 12 | [Don't Want to Lose Your Kids in the Divorce? Don't Be the Breadwinner](https://www.theguardian.com/women-in-leadership/2014/feb/10/female-breadwinners-lose-custody-divorce) | Google | The Guardian | England | Newspaper (Opinion) | 10-Feb-14 | Leans left | Marilyn Stowe |
| 13 | [Erick Erickson Is Terribly, Embarrassingly Wrong About Women](https://www.theatlantic.com/business/archive/2013/05/erick-erickson-is-terribly-embarrassingly-wrong-about-women/276410/) | Google | The Atlantic | USA | Magazine | 30-May-13 | Leans left | Derek Thompson |
| 14 | [Female Breadwinners Leading Households and Families More Than Ever](https://www.blackenterprise.com/female-breadwinners/) | Google | Black Enterprise | USA | Magazine | 08-Nov-16 | N/A | Carrie Pink |
| 15 | [Female Breadwinners Pay a Cost for Career Success – Marital Stress](https://www.theglobeandmail.com/report-on-business/careers/business-education/female-breadwinners-pay-a-cost-for-career-success-marital-stress/article35306676/) | Google | The Globe and Mail | Canada | Newspaper (Factual) | 14-Jun-17 | No bias | Darah Hansen |
| 16 | [Female Breadwinners: Why Earning More Can Poison your Marriage (but not in the way you'd expect)](https://www.telegraph.co.uk/women/11279824/Female-breadwinners-why-earning-more-can-poison-a-marriage.html) | Google | The Daily Telegraph | England | Newspaper (Factual) | 14-Dec-14 | Conservative | Julia Llewellyn Smith |
| 17 | [Four Couples Talk About What it’s Like When Women Earn More than Men](https://www.fastcompany.com/90248590/four-couples-talk-about-what-its-like-when-women-earn-more-than-men) | Google | Fast Company | USA | Magazine | 17-Oct-18 | Leans left | Pavithra Mohan |
| 18 | [Watch the Men of Fox News Freak Out Over Female Breadwinners](https://slate.com/human-interest/2013/05/four-out-of-ten-households-have-female-breadwinners-fox-news-responds-with-utter-panic.html) | Google | Slate | USA | Magazine | 30-May-13 | Liberal | Amanda Marcotte |
| 19 | [The Real Problem With Women as the Family Breadwinner](http://time.com/87890/fox-news-female-breadwinner/) | Google | Time | USA | Magazine | 05-May-14 | No bias | Belinda Luscombe |
| 20 | [What People Really Think About Working Moms](http://ideas.time.com/2013/05/31/what-people-really-think-about-working-moms/) | Google | Time | USA | Magazine | 31-May-13 | No bias | Judith Warner |
| 21 | [Husbands of Female Breadwinners Most at Risk for Cheating, Says Study](https://www.cnn.com/2016/10/07/health/infidelity-breadwinners-cheat-husband-wife/index.html) | Google | CNN | USA | News | 07-Oct-16 | No bias | Kelly Wallace |
| 22 | [Millennial Women Are Conflicted About Being Breadwinners](https://www.refinery29.com/en-us/2017/04/148488/millennial-women-are-conflicted-about-being-breadwinners) | Google | Refinery 29 | USA | Magazine | 01-May-17 | N/A | Ashley C. Ford |
| 23 | [Millennial Women are ‘Worried,’ ‘Ashamed’ of Out-Earning Boyfriends and Husbands](https://www.cnbc.com/2017/04/18/millennial-women-worry-about-out-earning-boyfriends-and-husbands.html) | Google | CNBC | USA | News | 19-Apr-17 | Conservative | Ester Bloom |
| 24 | [Moms Are Now Primary Breadwinners In 40 Percent of Homes](https://www.npr.org/sections/thetwo-way/2013/05/29/187019187/moms-are-now-primary-breadwinners-in-40-percent-of-homes) | Google | NPR | USA | News | 29-May-13 | Leans left | Mark Memmott |
| 25 | [Pew Study Shows Women Leading Breadwinners in 40 Percent of Households](https://www.thedailybeast.com/pew-study-shows-women-leading-breadwinners-in-40-percent-of-households) | Google | CNBC via Daily Beast | USA | News | 29-May-13 | Conservative | Amy Langfield |
| 26 | [Record Number of Female Breadwinners, According to Pew](https://abcnews.go.com/Business/record-number-female-primary-breadwinners-show-stark-differences/story?id=19280418) | Google | ABC News | USA | News | 29-May-13 | Leans left | Susanna Kim |
| 27 | [The 10 cities Where the Most Women Outearn Their Partners](https://www.cnbc.com/2019/03/08/the-10-cities-where-the-most-women-outearn-their-partners.html) | Google | CNBC | USA | News | 08-Mar-19 | Conservative | Kerri Anne Renzulli |
| 28 | [The Danger of Being a Breadwinning Wife](https://www.telegraph.co.uk/women/womens-life/11643189/The-danger-of-being-a-breadwinning-wife.html) | Google | The Daily Telegraph | England | Newspaper (Factual) | 01-Jun-15 | Conservative | Lucy Cavendish |
| 29 | [The Downside of Being the Breadwinner](https://www.newyorker.com/news/daily-comment/the-downside-of-being-the-breadwinner) | Google | The New Yorker | USA | Newspaper (Opinion) | 30-May-13 | Leans left | Margaret Talbot |
| 30 | [The Drawbacks of Being a Female Breadwinner](https://finance.yahoo.com/news/the-drawbacks-of-being-a-female-breadwinner-174853180.html) | Google | Yahoo Finance | USA | News | 25-Apr-14 | N/A | Farnoosh Torabi |
| 31 | [The Female Breadwinner Conundrum](https://thefederalist.com/2015/09/29/the-female-breadwinner-conundrum/) | Google | The Federalist | USA | Magazine | 29-Sep-15 | N/A | Joy Pullmann |
| 32 | [The Problem With 'Breadwinners'](https://psmag.com/economics/the-problem-with-breadwinners-64115) | Google | Pacific Standard | USA | Magazine | 14-Jun-17 | N/A | Philip N. Cohen |
| 33 | [U.S. Women on the Rise as Family Breadwinner](https://www.nytimes.com/2013/05/30/business/economy/women-as-family-breadwinner-on-the-rise-study-says.html) | Google | The New York Times | USA | Newspaper (Factual) | 29-May-13 | Leans left | Catherine Rampell |
| 34 | [What do Breadwinning Women Want? More Help, Less Stress](https://www.reuters.com/article/us-money-women-breadwinners/what-do-breadwinning-women-want-more-help-less-stress-idUSKCN0QP1MY20150820) | Google | Reuters | USA | News | 20-Aug-15 | Leans left | Lauren Young |
| 35 | [Life in the Only Industrialized Country Without Paid Maternity Leave](https://www.theatlantic.com/business/archive/2016/03/no-paid-maternity-leave/470889/) | Google | The Atlantic | USA | Magazine | 03-Mar-16 | Leans left | Jessica Shortall |
| 36 | [When She Earns More: As Roles Shift, Old Ideas on Who Pays the Bills Persist](https://www.nytimes.com/2018/07/06/your-money/marriage-men-women-finances.html) | Google | The New York Times | USA | Newspaper (Factual) | 06-Jul-18 | Leans left | Tara Siegel Bernard |
| 37 | [Why are Female Breadwinners Still 'Taboo'?](https://www.telegraph.co.uk/women/womens-business/9819500/Why-are-female-breadwinners-still-taboo.html) | Google | The Daily Telegraph | England | Newspaper (Opinion) | 23-Jan-13 | Conservative | Emma Sinclair |
| 38 | [Why You Shouldn't Feel Ashamed of Being a Female Breadwinner](https://www.bbc.co.uk/bbcthree/article/97983cce-dc31-4582-9434-74c84651267b) | Google | BBC | England | News | 31-May-18 | Leans left | Ashitha Nagesh |
| 39 | [When Women Make More, Couples Hide it](https://www.cnbc.com/2018/07/17/when-women-make-more-couples-hide-it.html) | Google | CNBC | USA | News | 18-Jul-18 | Conservative | Megan Leonhardt |
| 40 | [Switching 'Conventional' Gender Roles is Making Both Sexes Unhappy: Women Feel Depressed as Breadwinners as Are Men When They Are Stay-at-Home Fathers, Study Finds](https://www.dailymail.co.uk/health/article-4819356/Women-likely-feel-depressed-breadwinners.html) | Google | The Daily Mail | England | Newspaper (Factual) | 24-Aug-17 | Conservative | Claudia Tanner |
| 41 | [I Have a 6-figure Job and My Husband Stays Home With the Kids — Here are 10 Things No One Seems to Understand](https://www.businessinsider.com/working-mom-breadwinner-biggest-misconceptions-2018-7) | Google | Business Insider | USA | News | 14-Aug-18 | No bias | Liz Gendreau |
| 42 | 40 Per Cent of Women Earn More Than Their Man | Access | The Times | England | Newspaper (Factual) | 19-Jul-13 | Conservative | N/A |
| 43 | [A Recipe for Disaster](http://news.pioneergroup.com/manisteenews/2014/05/16/jim-crees-recipe-disaster/) | Access | Manistee News Advocate | USA | Newspaper (Opinion) | 16-May-14 | No bias | Jim Crees |
| 44 | [A Second Look at Millennials, Gender Roles](https://lacrossetribune.com/news/opinion/editorial/columnists/cynthia-m-allen-a-second-look-at-millennials-gender-roles/article_57d97e4b-fc7b-5331-8433-82237774f3b4.html) | Access | La Crosse Tribune | USA | Newspaper (Factual) | 28-Apr-17 | N/A | Cynthia M. Allen |
| 45 | [Women and Money: The Struggle to Juggle](https://www.ft.com/content/23e6713e-2a30-11e5-acfb-cbd2e1c81cca) | Access | Financial Times | England | Newspaper (Factual) | 17-Jul-15 | Leans right | Naomi Rovnick |
| 46 | [More U.S. Women Than Ever are Family Breadwinners, Pew Study Finds](https://www.latimes.com/nation/la-xpm-2013-may-28-la-na-breadwinner-moms-20130529-story.html) | Access | Los Angeles Times via The Herald (Rock Hill, SC) | USA | Newspaper (Factual) | 30-May-13 | Leans left | Emily Alpert |
| 47 | [America's Caregivers Deserve a Break](http://www.spokesman.com/stories/2013/aug/19/americas-caregivers-deserve-a-break/) | Access | Los Angeles Times via The Spokesman Review | USA | Newspaper (Opinion) | 19-Aug-13 | Leans left | N/A |
| 48 | [Back to the Kitchen, Guys. It's the Women Bringing Home the Bacon](https://www.thetimes.co.uk/article/back-to-the-kitchen-guys-its-the-women-bringing-home-the-bacon-50xcm5tgxgl) | Access | The Times | England | Newspaper (Factual) | 01-Jun-13 | Conservative | Devika Bhat |
| 49 | [Men Pay a Price in Poorer Health When Wives Earn More, Rutgers Study Finds](https://www.philly.com/philly/health/men-pay-a-price-in-poorer-health-when-wives-earn-more-rutgers-study-finds-20170921.html) | Access | Philadelphia Inquirer | USA | Newspaper (Factual) | 21-Sep-17 | Liberal | Stacey Burling |
| 50 | [Buck Up, Lads, UK Has 2m Do It All Women](https://www.thetimes.co.uk/article/buck-up-lads-uk-has-2m-do-it-all-women-8lpchf53w5s) | Access | The Times | England | Newspaper (Factual) | 04-Aug-13 | Conservative | Marie Woolf and Georgia Graham |
| 51 | [Teens Back Gender Equality in Business and Politics, but Not So Much in the Home](https://www.hoosiertimes.com/tmnews/life/teens-back-gender-equality-in-business-and-politics-but-not/article_5c3b9f9c-909b-5424-bb17-66129ea0d08c.html) | Access | Herald-Times | USA (Bloomington, IN) | Newspaper (Factual) | 01-Apr-17 | No bias | Lois M. Collins |
| 52 | [Couples Must Adjust When Wife is the Breadwinner](https://www.usatoday.com/story/money/personalfinance/2013/01/11/couples-adjust-women-breadwinner/1566254/) | Access | USA Today | USA | Newspaper (Factual) | 11-Jan-13 | No bias | Dana Hunsinger Benbow |
| 53 | [Data: Dads Making More Time for Kids](https://www.dailyprogress.com/news/data-dads-making-more-time-for-kids/article_4b5a2d0a-d631-11e2-aa37-0019bb30f31a.html) | Access | The Daily Progress | USA (Charlottesville, VA) | Newspaper (Factual) | 15-Jun-13 | N/A | J. Reynolds Hutchins |
| 54 | [Catherine Rampell: Hiring Women Can Boost the Bottom Line Because They’re Cheaper](https://www.washingtonpost.com/opinions/catherine-rampell-hiring-women-can-boost-the-bottom-line-because-theyre-cheaper/2014/04/03/58fe1e1a-bb4d-11e3-96ae-f2c36d2b1245_story.html) | Access | Charleston Gazette-Mail via Washington Post | USA (Charleston) | Newspaper (Opinion) | 08-Apr-14 | N/A | Catherine Rampell |
| 55 | [UCD Study: Gender Attitudes Chart Different Course Globally](https://www.davisenterprise.com/local-news/ucd/ucd-study-gender-attitudes-chart-different-course-globally/) | Access | The Davis Enterprise | USA (YOLO County) | Newspaper (Factual) | 21-Aug-18 | N/A | Kathleen Holder |
| 56 | [Equal pay for Equal Work Crosses Party Lines](https://helenair.com/news/opinion/equal-pay-for-equal-work-crosses-party-lines/article_60d16a22-0153-5817-a912-5ca8b1182f3a.html) | Access | The Independent Record | USA (Helena, MT) | Newspaper (Opinion) | 10-Feb-15 | No bias | Caitlin Copple |
| 57 | [Family: Female Breadwinners Are the New Norm, so Why Are We Still Doing All the Housework?](https://www.thetimes.co.uk/article/family-female-breadwinners-are-the-new-norm-so-why-are-we-still-doing-all-the-housework-pz7k8t7b7) | Access | The Times | England | Newspaper (Opinion) | 20-May-18 | Conservative | Lorraine Candy |
| 58 | [Female Business Ownership Climbs Nationally; Climate Strong Locally](https://www.staugustine.com/news/20180815/female-business-ownership-climbs-nationally-climate-strong-locally?template=ampart) | Access | St Augustine Record | USA (Florida) | Newspaper (Factual) | 15-Aug-18 | Leans right | Stuart Korfhage |
| 59 | [GSA Head Gwen Byrom: For a Full-Throttle Career, Girls, Get a Househusband](https://www.thetimes.co.uk/article/gsa-head-gwen-byrom-for-a-full-throttle-career-girls-get-a-househusband-0v8kjs29n) | Access | The Times | England | Newspaper (Factual) | 31-Dec-17 | Conservative | Sian Griffiths |
| 60 | [GENERATION Y: Why Worry if Women Make More Money?](https://www.stwnewspress.com/opinion/generation-y-why-worry-if-women-make-more-money/article_8b89991a-6394-11e8-9afb-1b39975994e1.html) | Access | Stillwater News Press | USA (OK) | Newspaper (Opinion) | 30-May-18 | N/A | Kieran Steckley |
| 61 | [Rozner: Golfer Stacy Lewis Scores Huge Cictory for Women](https://www.dailyherald.com/sports/20180630/rozner-golfer-stacy-lewis-scores-huge-victory-for-women-of-lpga) | Access | The Daily Herald | USA (Arlington Heights, IL) | Newspaper (Factual) | 01-Jul-18 | N/A | Barry Rozner |
| 62 | [When She Makes More' Offers Tips For Female Breadwinners](https://www.theledger.com/business/20140518/when-she-makes-more-offers-tips-for-female-breadwinners) | Access | The Ledger | USA (Lakeland, FL) | Newspaper (Factual) | 18-May-14 | N/A | Tim Grant |
| 63 | [Guys, Take Pride in Your Alpha Wives](https://www.thetimes.co.uk/article/guys-take-pride-in-your-alpha-wives-xmc8wsl2tc8) | Access | The Times | England | Newspaper (Opinion) | 22-Dec-13 | Conservative | Eleanor Mills |
| 64 | [Have a Problem with Women as Breadwinner? Get….](https://www.newsday.com/opinion/oped/who-cares-if-women-are-the-breadwinners-demetria-l-lucas-1.5407856) | Access | Fort Worth Star-Tellegram adapted from The Root | USA (TX) | Newspaper (Opinion) | 06-Jun-13 | Liberal | Demetria L. Lucas |
| 65 | Have These Men Lost Their Minds | Access | South Jersey times | USA (NJ) | Newspaper (Opinion) | 20-Jun-13 | N/A | Cokie Roberts and Steven Roberts |
| 66 | [In Our Opinion: Equal Pay Still Eludes Female Workers](https://www.thedailystar.com/opinion/in-our-opinion-equal-pay-still-eludes-female-workers/article_f8de9b3c-461b-54eb-b84e-7a2f136fbb80.html) | Access | The Daily Star | USA (Oneonta, NY) | Newspaper (Opinion) | 09-Apr-14 | N/A | N/A |
| 67 | [Stay-at-Home Dads? Why Swapping Gender Roles Doesn't Always Work](https://www.telegraph.co.uk/men/relationships/fatherhood/11242851/Stay-at-home-dads-Why-swapping-gender-roles-doesnt-always-work.html) | Access | The Daily Telegraph | England | Newspaper (Opinion) | 22-Nov-14 | Conservative | Becky Dickinson |
| 68 | Many Women Now Breadwinner Moms | Access | Madera Tribune | USA (CA) | Newspaper (Opinion) | 06-Jun-13 | N/A | Jim Glynn Howling |
| 69 | [The Secret of a Happy Marriage: Women Wearing the Trousers](https://www.telegraph.co.uk/women/sex/divorce/10644125/The-secret-of-a-happy-marriage-women-wearing-the-trousers.html) | Access | The Daily Telegraph | England | Newspaper (Factual) | 18-Feb-14 | Conservative | John Bingham |
| 70 | [Decline in Traditionally Male Industries, Economic Austerity and Better Opportunities for Women Mean More and More Mums Bring Home the Bacon](https://www.independent.co.uk/life-style/health-and-families/health-news/decline-in-traditionally-male-industries-economic-austerity-and-better-opportunities-for-women-mean-8744980.html) | Access | The Independent | England | Newspaper (Factual) | 04-Aug-13 | Leans left | Jane Merrick |
| 71 | [More Fathers Stay at Home to Raise Children](https://www.staugustine.com/article/20130616/NEWS/306169954) | Access | St Augustine Record | USA (FL) | Newspaper (Factual) | 16-Jun-13 | Leans right | Sheldon Gardner |
| 72 | More Female Heads of Household a Good Sign | Access | Elkhart Truth | USA (IN) | Newspaper (Factual) | 01-Jun-13 | N/A | Joe Heller |
| 73 | Pay Gap for Women Bigger in Denton County Pay Gap | Access | Denton Record-Chronicle | USA (TX) | Newspaper (Factual) | 04-Feb-18 | N/A | Peggy Heinkel-Wolfe |
| 74 | Rise of the Female Breadwinner | Access | Financial Times | England | Newspaper (Factual) | 18-Jul-15 | Leans right | N/A |
| 75 | [Being Sole Breadwinner is Bad for Men’s Health but Good for Women](https://www.telegraph.co.uk/science/2016/08/19/being-sole-breadwinner-is-bad-for-mens-health-but-good-for-women/) | Access | The Daily Telegraph | England | Newspaper (Factual) | 19-Aug-16 | Conservative | Sarah Knapton |
| 76 | Risk of Divorce Higher for Husbands Who Stay at… | Access | The Daily Telegraph | England | Newspaper (Factual) | 28-Jul-16 | Conservative | N/A |
| 77 | [Don't Forget Men in the Shifts that are Reshaping Society](https://www.theguardian.com/commentisfree/2013/aug/04/gender-men-need-space-to-debate-new-roles) | Access | The Observer | England | Newspaper (Opinion) | 04-Aug-13 | N/A | Yvonne Roberts |
| 78 | [Household Earnings: Local Women Contribute to 'Primary Breadwinner' Statistic](https://www.aikenstandard.com/news/household-earnings-local-women-contribute-to-primary-breadwinner-statistic/article_c8a73b89-c274-539d-a8fd-eadb47313a78.html) | Access | Aiken Standard | USA (SC) | Newspaper (Factual) | 06-Jul-13 | Leans right | Katie Binion |
| 79 | Study Shows Increasing Female Breadwinners - But… | Access | The Gazette | USA (IA) | Newspaper (Factual) | 30-May-13 | No bias | Kiran Sood |
| 80 | Tackling the Female Financial Paradox in…. | Access | The Birmingham Post | England | Newspaper (Opinion) | 01-May-14 | N/A | Lisa Johnson |
| 81 | [Motherhood Gap Leaves Moms with Lower Pay, More Stress](https://www.timesonline.com/article/20160508/News/305089932) | Access | The Allegheny Times | USA (Beaver, PA) | Newspaper (Factual) | 08-May-16 | No bias | Daveen Rae Kurutz |
| 82 | [Breadwinner Moms Change the Face of American Life](https://www.google.com/url?sa=t&rct=j&q=&esrc=s&source=web&cd=1&cad=rja&uact=8&ved=2ahUKEwj5kJiS4KviAhXkIzQIHTCUDNEQFjAAegQIBBAB&url=https%3A%2F%2Fwww.pressreader.com%2Fcanada%2Fottawa-citizen%2F20130603%2F282205123432567&usg=AOvVaw08K_Rn1sY7aiKvr0x7-jGu) | Access | The Daily Telegraph | England | Newspaper (Factual) | 20-May-13 | Conservative | Philip Sherwell |
| 83 | The Image of a Modern Woman | Access | The Independent | England | Newspaper (Factual) | 13-Mar-16 | Leans left | Joanna Moorhead |
| 84 | [Growing Number of Women Out-earn Husbands](https://archive.triblive.com/lifestyles/more-lifestyles/growing-number-of-women-out-earn-husbands/) | Access | Pittsburgh Tribune-Review | USA | Newspaper (Factual) | 06-Apr-14 | Conservative | Rachel Weaver |
| 85 | [What She Said: Lisa Michelle Borders, Time’s Up CEO, Answers your Work-Related Dilemma](https://www.thetimes.co.uk/article/what-she-said-lisa-michelle-borders-time-s-up-ceo-answers-your-work-related-dilemma-xdrcrr28s) | Access | The Sunday Times | England | Newspaper (Opinion) | 20-Jan-19 | Conservative | Fleur Britten |
| 86 | [How Being a Kept Man Could Raise your Heart Attack Risk](https://www.thetimes.co.uk/article/men-who-lose-position-as-chief-breadwinner-increase-risk-of-heart-attacks-diabetes-and-stroke-say-team-from-rutgers-university-new-jersey-q9zxt57jr) | Access | The Times | England | Newspaper (Factual) | 05-Sep-17 | Conservative | Tom Whipple |
| 87 | [Husbands Happiest When they Earn Half as Much as Wives](https://www.thetimes.co.uk/article/husbands-happiest-when-they-earn-half-as-much-as-wives-z5s66q20z) | Access | The Times | England | Newspaper (Factual) | 19-Aug-16 | Conservative | Tom Whipple |
| 88 | [Why America is Waiting to Get Married (Hint: It's not because we don't value relationships)](https://www.deseretnews.com/article/865612771/Why-America-is-waiting-to-get-married-Hint-Its-not-because-we-dont-value-relationships.html) | Access | Deseret News | USA (Salt Lake City, Utah) | Newspaper (Factual) | 09-Oct-14 | Conservative | Herb Scribner |
| 89 | Why are More Women Divorced | Access | Air Force Times | USA | Newspaper (Factual) | 05-May-14 | N/A | N/A |
| 90 | Women on the Move… | Access | The Register-Guard | USA (Eugene, OR) | Newspaper (Factual) | 04-Jun-13 | No bias | N/A |
| 91 | [Women in the U.S. Still Do Way More Housework Than Men](https://www.bloomberg.com/news/articles/2015-06-26/women-in-the-u-s-still-do-way-more-housework-than-men) | Access | Bloomberg News via Bay City Times | USA (MI) | Newspaper (Factual) | 06-Jul-15 | N/A | Sheelah Kolhatkar |
| 92 | [Women the Main Breadwinners in Record 40% of US Homes](https://www.ft.com/content/7a185746-c869-11e2-acc6-00144feab7de) | Access | Financial Times | England | Newspaper (Factual) | 29-May-13 | Leans right | Norma Cohen |
| 93 | Work Demands Rising for Moms, but Not Pay | Access | Dayton Daily News | USA (OH) | Newspaper (Factual) | 08-May-16 | Leans right | Randy Tucker |
| 94 | [Our View: More Female Breadwinners](https://www.standard.net/opinion/our-view-more-female-breadwinners/article_02a857de-1e73-58ea-8ea2-b93b0adfb8a7.html) | Access | Standard-Examiner | USA (Ogden, UT) | Newspaper (Opinion) | 10-Jun-13 | No bias | N/A |

**Coding Article Descriptives**

***Type of Article***

The type of article was categorized as magazine (online only or online version of a print magazine; e.g., *Refinery29*), online news source (e.g., *CNBC*), or online version of a print newspaper (e.g., *The New York Ti*mes). The online version of a print newspaper was further categorized as a factual article, defined as an article that reports results from studies, other facts, expert testimonials, or quotes from first-person experiences, but does not express the writer’s opinion on this information; or an opinion article, defined as an article that may contain some or all of the elements of factual newspaper articles, but which also includes the writer’s opinion.

***Political Lean***

Following previous research in this area (Gentzkow & Shapiro, 2010), we used *Mondo Times* (n.d.) to obtain ratings of the political lean of the sampled articles, which is a media directory website that provides the political leaning of various American, Canadian, and international news sources based on users’ votes.

**Descriptive Results**

Seventy-one percent of the articles were from American sources, one percent Canadian, and 28% were from the UK. Of these articles, 85% were from news sources or newspapers. Specifically, 47% of all articles were factual newspaper articles, 21% of all articles were opinion newspaper articles, and 17% of all articles were from news sources. Fifteen percent of all articles were from magazines. The articles that composed this sample came from both large-scale sources (e.g., *The New York Post*, *Forbes*, *The Daily Telegraph*) as well as smaller-scale sources (e.g., *Stillwater* *News Press*, *The Allegheny Times*). We were concerned that only one article was Canadian. This was not a sampling error, as Access World News does search Canadian sources – both large-scale (e.g., *CBC*, *The Toronto Star*) and smaller-scale (e.g., *The Guelph Tribune*, *Langley Times*). Furthermore, there were some Canadian sources present in the Google search results as well. Canada may have fewer female breadwinner articles because it is a strong social democracy (Wiseman & Isitt, 2007) with a relatively weak populist movement compared to the UK and the US, and thus female breadwinners are less threatening to Canadian social norms than to norms in the UK and the US. This may have resulted in less Canadian news content regarding female breadwinners compared to American and English news content.

Article dates of publication ranged from January 11, 2013 to March 8, 2019. The majority of articles were written in 2013 (37%), followed by 2014 (17%), 2017 and 2018 (both 13%), 2016 (11%), 2015 (7%), and 2019 (2%).

Thirty-five percent of all articles were from Conservative or “right-leaning” news sources, 21% of all articles were from Liberal or “left-leaning” news sources, 15% of all articles were from “central” or “no-bias” news sources, and 29% of all articles come from news sources with unknown political leanings.

Citations not included in the manuscript:

Mondo Times (n.d.). *Find News media Worldwide.* https://www.mondotimes.com/

**Studies 2a and 2b**

**Notes About the Pre-Registration**

Reviewers were informed about these notes in the manuscript during review.

In the paper, we described that we predicted that compared to the male breadwinner couple, participants would perceive that the female breadwinner couple experiences worse relationship quality and more gender threat (i.e., greater feelings of gender nonconformity and inadequacy). Our pre-registration framed these predictions as greater ‘costs’ (i.e., gender nonconformity, inadequacy) and fewer ‘benefits’ (i.e., relationship quality) in the FBR than the MBR.

Our pre-registration also included the prediction that participants who more strongly endorsed traditional gender-role attitudes would express more negative evaluations in the FBR condition, but this hypothesis was generally not supported; gender attitudes did not moderate any of the results we report except for perceptions of gender nonconformity in Study 2a. More details are offered below.

**Study 2a**

**Additional Measures**

***Individual Difference Variables***

**Political Orientation.** Participants indicated how they would describe themselves politically using a 7-point scale (1 – left-wing, 7 – right wing).

**Self-Esteem.** Participants used a 7-point Likert scale (1 – strongly disagree, 7 – strongly agree) to rate their agreement with the statement, “I have high self-esteem.”

**Gender Role Attitudes.** Participants used a 7-point Likert-type scale (1 – strongly disagree, 7 – strongly agree) to indicate their agreement with 10-items assessing their gender-role attitudes (GRA; adapted from Larsen & Long, 1988; e.g., “Women should have as much sexual freedom as men;” “The man should be more responsible for the economic support of the family than the woman”). Items were coded such that higher scores indicated more egalitarian attitudes and then averaged to form a reliable index of *GRA* (α = .89).

**Personality.** Participants completed the Ten Item Personality Inventory (TIPI; Gosling et al., 2003).

***Dependent Variables***

**Domestic Work.** Participants again used a 7-point scale (1 – strongly disagree, 7 – strongly agree) to answer three items assessing how much domestic work the target person would perform (e.g., “She/he would be more responsible for managing the home than her/his partner” and “She/he would complete more housework (e.g., chores, childcare, and errands) than his partner”).

**Financial Responsibility.** Participants used the same 7-point scale to answer three items assessing the perceived financial responsibility of each partner in the relationship (e.g., She/he would be more responsible for paying the bills than her partner.

**Relationship Work.** Participants used the same 7-point scale to answer three items assessing who was responsible for doing more relationship work (e.g., “She/he would be less responsible for maintaining the relationship than her/his partner”).

**Conflict.** Participants used the same scale to answer four items about conflict in the target’s marriage (e.g., “She/he would rarely argue with her/his partner;” “She/he would often find reasons to start a fight or argument with her/his partner).

**Gender Role Attitudes Analyses**

Gender role attitudes moderated only one of the outcomes we report: perceived gender nonconformity. Both participants with more egalitarian and more traditional gender role attitudes thought that Zach was more gender non-conforming in the FBR than the MBR, but this effect was stronger for egalitarian people, who thought Zach in the MBR was especially gender conforming.

# **Study 2b**

**Additional Measures in Study 2b**

***Gender Role Attitudes.*** Same as in Study 2a. Higher scores indicated more egalitarian attitudes.

***Political Ideology.*** Participants indicated how they would describe themselves politically using a 7-point scale (1 – left-wing, 7 – right wing).

***Domestic Work.*** Same as Study 2a.

***Financial Responsibility.*** Same as Study 2a.

***Relationship Work****.* Same as Study 2a.

***Conflict*.** Same as Study 2a.

***Cheat.*** Participants used a 7-point scale (1 – strongly disagree, 7 – strongly agree) to answer two items about the likelihood of cheating in the marriage (e.g., “She/he would be more likely to "cheat" on his/her partner;” “Her/his partner would be more likely to "cheat" on her/him).

**Gender Role Attitudes Analyses**

Gender role attitudes did not moderate any of the outcomes we considered.

**Exploratory Analysis for Moderation by Sexual Orientation Across Studies 2a and 2b**

As per reviewer request, we explored whether our experimental results were moderated by participant sexual orientation (roughly dichotomized into straight/heterosexual and LGBTQIA+ groupings). None of the results in Study 2a were moderated by sexual orientation. However, all of the results of Study 2b were moderated by sexual orientation albeit the sample size of LGBTQIA+ (n = ~177) to straight/heterosexual participants (n=~1000) was highly unequal. The results for straight participants mirrored our reported results whereas the results for LGBTQIA+ participants differed. For relationship quality and feelings of inadequacy, there were no effects for LGBTQIA+ participants. For relationship dissolution, there was an interaction effect for LGBTQIA+ participants (compared to a main effect of relationship type for straight participants) such that they perceived Olivia as more likely to have her relationship end in the FBR than the MBR. For social approval, there was an interaction for LGBTQIA+ participants (vs main effect of relationship type for straight participants) whereby they perceived Olivia as receiving less social approval in the FBR than the MBR. For gender nonconformity there was only a main effect of relationship type for LGBTQIA+ participants (vs the anticipated interaction for straight participants). They saw more gender nonconformity in the FBR compared to the MBR. In sum, compared to straight participants, LGBTQIA+ participants perceived unique individual consequences for Olivia relating to relationship dissolution and social approval but collective relationship-level consequences for gender nonconformity.

# **Study 3**

**Information Regarding the Coding Error for High Incomes**

Unfortunately, the data for participants who reported household incomes greater than $14k could not be used to obtain reliable results. The income measure used $1k increments up to $7k per month (ie.., 3 = $2000 to $3000, 4 = $3000 to $4000… 7 = $6000 to $7000) but a coding error resulted in the scale skipping two increments for incomes greater than $7k per month (i.e., 8 = $8000 to $9000, 9 = $10000 and over). This introduced two forms of unreliability. First, we cannot be certain how people with scores in the missing increments used the scale (did they choose the lower or higher value?). And second, the calculation of the key variable in our model – female proportion of income earned – would be highly imprecise for people with scores in that range, especially if both partners had incomes higher that $7k (worst case could be a $4k inaccuracy if they chose a lower option for one partner and a higher option for the other for incomes in the missing increments). For these reasons we opted to exclude these participants from our study. Fortunately, we had a very large sample and have adequate power even after that exclusion.

This decision yielded a few side benefits. The resulting sample has an average income very close to the median household income in the US per the 2020 US census, and the resulting distribution of scores is normal (neither of these are the case for the full sample). It may also be important to note that from an ethical perspective, these participants’ data have not been wasted because they were included in a completely unrelated paper about the prevalence of friends-first relationship initiation (as reported in the manuscript).

**Additional Measures**

***Demographics***

1. What is your relationship status? (Options: married or common law)
2. What is the highest degree or level of schooling/education you have completed? *If currently enrolled, highest degree received.*

Options:

- No schooling completed
- Elementary to 8^th^ grade
- Some high school, no diploma
- High school graduate, diploma or equivalent (e.g., GED)
- Some college credit, no degree
- Trade/technical/vocational training
- Associate degree/diploma
- Bachelor’s degree
- Master’s degree
- Professional degree
- Doctorate degree

1. What is the highest degree or level of schooling/education your partner has completed? *If currently enrolled, highest degree received.*

Options:

- No schooling completed
- Elementary to 8^th^ grade
- Some high school, no diploma
- High school graduate, diploma or equivalent (e.g., GED)
- Some college credit, no degree
- Trade/technical/vocational training
- Associate degree/diploma
- Bachelor’s degree
- Master’s degree
- Professional degree
- Doctorate degree

1. How many children primarily live in your household (i.e., primary custody; 60% or more of the child’s time)? (drop down; options 1 through 8)
2. How many children in your household are between 0-12 years? (options 1 through 8)
3. How many children in your household are between 13-18? (options 1 through 8)
4. How many people in your household are over the age of 18? (options 1 through 10)
5. Does anyone in your household require additional support (e.g., child or family member with a disability, older family members, etc.) (Yes/No)
6. What city/town do you currently live in? (Fill in blank)
7. How would you describe yourself politically? (1 = extremely liberal, 7 = extremely conservative)

***Socioeconomic Status***

**Imagine that this ladder shows how your society is set up.** At the top of the ladder are the people who are **best off** – they have the most money, the highest amount of schooling, and the jobs that bring the most respect. At the bottom are the people who are the **worst off** – they have the least money, little or no education, no jobs or jobs that no one wants or respects.

**Now think about yourself. Please tell us where you think you would be on this ladder. Select the number that best represents where you would be on this ladder.**


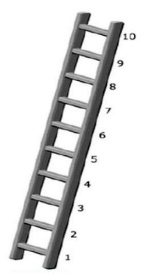


1. My current employment situation is best described as . . .

(Options: Employed, at work; Employed, working from home; Employed, absent from work; Temporary layoff; Job seeker; Future start; Not in labour force, able to work; Not in labour force, permanently unable to work, N/A) *Fill in option

1. My current occupation is best described as . . . (Options: Management occupations; Business, finance and administration occupations; Natural and applied sciences and related occupations; Health occupations; Occupations in education, law and social, community and government services; Occupations in art, culture, recreation and sport; Sales and service occupations; Trades, transport and equipment operators and related occupations; Natural resources, agriculture and related production occupations; Occupations in manufacturing and utilities, N/A) *Fill in option
2. Approximately how many hours **per week** do you engage in paid work? (Drop Down: less than 10; 10 to 20; 20 to 30; 30 to 40; more than 40; N/A)
3. Approximately how many hours of unpaid household labour do you engage in **on an average weekday**? (E.g., meal preparation, yard work, house cleaning, shopping for groceries, etc.) (Drop down: I do not do housework; 1 – less than 1 hour, 6 – 8 to 10 hours, 11 – more than 18 hours)
4. Approximately how many hours **per day** do you spend taking care of your child(ren) on an average weekday? (E.g., helping with homework, looking after, playing, booking appointments or activities, etc.)
5. What is your spouse or common-law partner’s current employment situation best described as . . . (Options: Employed, at work; Employed, working from home; Employed, absent from work; Temporary layoff; Job seeker; Future start; Not in labour force, able to work; Not in labour force, permanently unable to work, N/A) * Fill in option
6. What is your spouse or common-law partner’s current occupation? (Options: Management occupations; Business, finance and administration occupations; Natural and applied sciences and related occupations; Health occupations; Occupations in education, law and social, community and government services; Occupations in art, culture, recreation and sport; Sales and service occupations; Trades, transport and equipment operators and related occupations; Natural resources, agriculture and related production occupations; Occupations in manufacturing and utilities, N/A) *Fill in option
7. Approximately how many hours **per week** does **your partner** engage in paid work? (Drop Down: less than 10; 10 to 20; 20 to 30; 30 to 40; more than 40; N/A)
8. Approximately how many hours of unpaid household labour does **your partner** you engage in **on an average weekday**? (E.g., meal preparation, yard work, house cleaning, shopping for groceries, etc.) (Drop down: less than 5; 5 to 10; 10 to 15; 15 to 20; 25 to 30; 30 to 35; 35 to 40; more than 40; N/A)
9. Approximately how many hours **per day** does **your partner** spend taking care of your child(ren) on an average weekday? (E.g., helping with homework, looking after, playing, booking appointments or activities, etc.)
10. Approximately how many hours per week does your child receive care from another person? (E.g., the child’s biological parent, grandparents, neighbours, etc.) (Drop down: less than 5; 5 to 10; 10 to 15; 15 to 20; 25 to 30; 30 to 35; 35 to 40; more than 40; N/A)
11. What is your child’s current schooling/childcare situation best described as? (Drop down; Full time school/childcare; Part time school/childcare; Full time homeschooling/no childcare)

These questions ask about your work and home life before the COVID pandemic:

1. What was your employment status ***before*** the COVID-19 pandemic? (Options: Employed, at work; Employed, absent from work; Temporary layoff; Job seeker; Future start; Not in labour force, able to work; Not in labour force, permanently unable to work, N/A)
2. What was your occupation ***before*** the COVID-19 pandemic? (Options: Management occupations; Business, finance and administration occupations; Natural and applied sciences and related occupations; Health occupations; Occupations in education, law and social, community and government services; Occupations in art, culture, recreation and sport; Sales and service occupations; Trades, transport and equipment operators and related occupations; Natural resources, agriculture and related production occupations; Occupations in manufacturing and utilities, N/A) * Fill in option
3. What was your **approximate monthly income *before*** COVID-19, including public assistance from other sources but **NOT** including your spouse or common-law partner’s income? (Options: N/A; Income under $1000; Income from $1000 to $2000; Income from $2000 to $3000; Income from $3000 to $4000; Income from $4000 to $5000; Income from $5000 to $6000; Income from $6000 to $7000; Income from $8000 to $9000; Income from $10,000 and over)
4. Approximately how many hours **per week** did you engage in paid work ***before*** COVID-19? (Drop Down: less than 10; 10 to 20; 20 to 30; 30 to 40; more than 40; N/A)
5. Approximately how many hours of unpaid household labour did you engage in **on an average weekday *before*** COVID? (E.g., meal preparation, yard work, house cleaning, shopping for groceries, etc.) (Drop down: less than 5; 5 to 10; 10 to 15; 15 to 20; 25 to 30; 30 to 35; 35 to 40; more than 40; N/A)
6. Approximately how many hours **per day** did you spend taking care of your child(ren) on an average weekday ***before*** COVID-19? (E.g., helping with homework, looking after, playing, booking appointments or activities, etc.) (Drop down: less than 5; 5 to 10; 10 to 15; 15 to 20; 25 to 30; 30 to 35; 35 to 40; more than 40; N/A)

These questions ask about your spouse or common-law partner’s work and home life before the COVID pandemic

1. What was your spouse or common-law partner’s employment status ***before*** the COVID-19 pandemic? (Options: Employed, at work; Employed, absent from work; Temporary layoff; Job seeker; Future start; Not in labour force, able to work; Not in labour force, permanently unable to work, N/A) If other, please specify __________
2. What was your spouse or common-law partner’s occupation ***before*** the COVID-19 pandemic? (Options: Management occupations; Business, finance and administration occupations; Natural and applied sciences and related occupations; Health occupations; Occupations in education, law and social, community and government services; Occupations in art, culture, recreation and sport; Sales and service occupations; Trades, transport and equipment operators and related occupations; Natural resources, agriculture and related production occupations; Occupations in manufacturing and utilities, N/A) * Fill in option
3. What was **your partner’s approximate monthly income *before*** COVID-19, including public assistance from other sources but **NOT** including your spouse or common-law partner’s income? (Options: N/A; Income under $1000; Income from $1000 to $2000; Income from $2000 to $3000; Income from $3000 to $4000; Income from $4000 to $5000; Income from $5000 to $6000; Income from $6000 to $7000; Income from $8000 to $9000; Income from $10,000 and over)
4. Approximately how many hours **per week** did **your partner** engage in paid work ***before*** COVID-19? (Drop Down: less than 10; 10 to 20; 20 to 30; 30 to 40; more than 40; N/A)
5. Approximately how many hours of unpaid household labour did **your partner** engage in **on an average weekday *before*** COVID? (E.g., meal preparation, yard work, house cleaning, shopping for groceries, etc.) (Drop down: less than 5; 5 to 10; 10 to 15; 15 to 20; 25 to 30; 30 to 35; 35 to 40; more than 40; N/A)
6. Approximately how many hours **per day** did **your partner** spend taking care of your child(ren) on an average weekday ***before*** COVID-19? (E.g., helping with homework, looking after, playing, booking appointments or activities, etc.) (Drop down: less than 5; 5 to 10; 10 to 15; 15 to 20; 25 to 30; 30 to 35; 35 to 40; more than 40; N/A)
7. How many hours per week did your child receive care from another person (e.g., the child’s biological parent, grandparents, neighbours, childcare provider etc.) ***before*** COVID-19? (Drop down: less than 5; 5 to 10; 10 to 15; 15 to 20; 25 to 30; 30 to 35; 35 to 40; more than 40; N/A)
8. What was your child’s schooling/childcare situation best described as ***before*** COVID-19? (Drop down; Full time school/childcare; Part time school/childcare; Full time homeschooling/no childcare, N/A)

***Covid Stress***

1. In the last month, how often have you felt confident about your ability to handle your personal problems?
2. In the last month, how often have you felt that things were going your way?
3. In the last month, how often have you felt difficulties were piling up so high that you could not overcome them?
4. In the last month, how often have you felt that concerned that yourself or one of your loved ones would come down with the COVID-19 virus?
5. In the last month, how often have you felt anxious about the impacts of the COVID-19 pandemic on your family and your community?
6. In the past month, how often have you been concerned about the unexpected financial impacts of COVID-19?
7. In the last month, how often have you felt unable to control the important things in your life?

***Self-Esteem***

1. I have high self-esteem

***Financial Equity/Fairness***

1. I feel pressure to provide financially
2. My partner treats me to luxuries I cannot afford
3. I feel a sense of independence in my relationship
4. I make decisions in my relationship
5. My opinions matter more than my spouse or common-law partner’s opinion
6. I have access to resources (e.g., healthcare, transportation, etc.) that improve my quality of life
7. I have time for activities and hobbies outside of work
8. I feel my partner is contributing enough to the relationship

***Conflict***

1. I rarely argue with my spouse or common-law partner
2. I often find reasons to start a fight or argument with my spouse or common-law partner
3. I feel resentful towards my spouse or common-law partner
4. I get along well with my spouse or common-law partner

***Dependence Promotion***

1. I searched for something my partner had lost (e.g., keys, glasses, umbrella)
2. I listened carefully to something my partner wanted to talk about (e.g., work, sports, another person) even though I had little or no interest on the issue
3. I cleaned up a mess of some sort (e.g., clothes on the floor, dirty dishes, something which had broken or spilled) for my partner
4. I repaired something my partner had damaged or broken
5. I bought and gave a nice gift to my partner
6. I took time out of my busy day to do an errand or chore for my partner
7. I waited for my partner to finish something even though I was anxious to go somewhere or do something
8. I did a chore that is usually my partner’s responsibility so they could relax
9. I cooked my partner their favorite meal
10. I looked after the children so my partner could spend time with friends or participate in a hobby
11. I offered my partner emotional support for a problem they were having
12. I cared for our children so my partner could relax
13. I resisted talking about a personal problem to not burden my partner
14. I organized my child’s appointments, extracurricular activities or childcare/school administration so my partner didn’t have to worry about it
15. I monitored my child(ren) for COVID-19 symptoms so that my partner didn’t have to
16. I gave up some time that I planned for myself to accommodate my partner’s needs

***Division of Household Labour***

1. Cleaning and laundry
2. Meal preparation
3. Yard work
4. Homeschooling or helping children with schoolwork
5. Vehicle maintenance
6. Childcare (e.g., feeding, bathing)
7. Providing emotional support for family members (e.g., soothing children, settling disputes etc.)
8. Family organization and planning (e.g., making appointments, ensuring tasks get completed etc.)
9. Paying bills and managing finances
10. Planning family leisure time (e.g., planning dates, family outings)
11. Transporting children to school and activities
12. Taking out the trash/garbage
13. Home repairs and fixing things around the house

**Supplemental Analyses for Same Sex/Gender and Queer Relationships**

| **Table S2**.  *Descriptive statistics and correlations among study variables for participants in a relationship where both partners are the same sex/gender or at least one partner is trans/non-binary (n = 35)* | | | | | | | | | |
| --- | --- | --- | --- | --- | --- | --- | --- | --- | --- |
| Variable | *M* | *SD* | 2 | 3 | 4 | 5 | 6 | 7 |  |
| 1. Partner’s POI | 0.47 | 0.14 | .14 | .31 | .10 | .42* | .49** | -.25 |  |
| 2. Political Ideology | 5.00 | 2.00 | - | .51** | .16 | .39* | .30 | .11 |  |
| 3. Femininity | 5.20 | 1.85 |  | - | -.01 | .72** | .66** | .12 |  |
| 4. Masculinity | 5.37 | 1.75 |  | - | - | .35* | .35* | .33 |  |
| 5. Inadequacy | 4.84 | 1.92 |  |  | - | - | .92** | -.14 |  |
| 6. Conflict | 3.90 | 1.41 |  |  |  | - | - | -.24 |  |
| 7. Relationship Quality | 5.98 | 0.76 |  |  |  |  |  | - |  |
| *Note.* **p* < .05; ***p* < .01; POI = proportion of income | | | | | | | | | |

We included partner’s proportion of income earned in this correlation matrix instead of the female partner’s proportion of income, which we report in the main manuscript, because the latter is not relevant for many LGBTQ+ relationships.

**Full PROCESS Results**

Run MATRIX procedure:

***************** PROCESS Procedure for SPSS Version 4.0 *****************

Written by Andrew F. Hayes, Ph.D. www.afhayes.com

Documentation available in Hayes (2022). www.guilford.com/p/hayes3

**************************************************************************

Model : 91

Y : QComp

X : W_POIC

M1 : GDcent

M2 : InadCent

W : P_GenBin

Sample

Size: 508

**************************************************************************

OUTCOME VARIABLE:

GDcent

Model Summary

R R-sq MSE F df1 df2 p

.1739 .0302 3.5247 15.7775 1.0000 506.0000 .0001

Model

coeff se t p LLCI ULCI

constant .0147 .0833 .1769 .8596 -.1490 .1785

W_POIC 1.5799 .3977 3.9721 .0001 .7985 2.3613

**************************************************************************

OUTCOME VARIABLE:

InadCent

Model Summary

R R-sq MSE F df1 df2 p

.7428 .5517 1.1232 154.7833 4.0000 503.0000 .0000

Model

coeff se t p LLCI ULCI

constant -.0154 .0632 -.2441 .8073 -.1395 .1087

W_POIC -.1583 .2333 -.6783 .4979 -.6167 .3001

GDcent .5324 .0351 15.1841 .0000 .4635 .6013

P_GenBin .0214 .0971 .2207 .8254 -.1693 .2121

Int_1 .1617 .0495 3.2681 .0012 .0645 .2588

Product terms key:

Int_1 : GDcent x P_GenBin

Test(s) of highest order unconditional interaction(s):

R2-chng F df1 df2 p

M1*W .0095 10.6802 1.0000 503.0000 .0012

----------

Focal predict: GDcent (M1)

Mod var: P_GenBin (W)

Conditional effects of the focal predictor at values of the moderator(s):

P_GenBin Effect se t p LLCI ULCI

.0000 .5324 .0351 15.1841 .0000 .4635 .6013

1.0000 .6940 .0355 19.5471 .0000 .6243 .7638

**************************************************************************

OUTCOME VARIABLE:

QComp

Model Summary

R R-sq MSE F df1 df2 p

.4141 .1715 .9657 34.7717 3.0000 504.0000 .0000

Model

coeff se t p LLCI ULCI

constant 5.8927 .0436 135.0963 .0000 5.8070 5.9784

W_POIC -.4808 .2115 -2.2728 .0235 -.8964 -.0652

GDcent .0497 .0342 1.4529 .1469 -.0175 .1169

InadCent -.3112 .0409 -7.6084 .0000 -.3916 -.2309

****************** DIRECT AND INDIRECT EFFECTS OF X ON Y *****************

Direct effect of X on Y

Effect se t p LLCI ULCI

-.4808 .2115 -2.2728 .0235 -.8964 -.0652

Conditional and unconditional indirect effects of X on Y:

INDIRECT EFFECT:

W_POIC -> GDcent -> QComp

Effect BootSE BootLLCI BootULCI

.0785 .0770 -.0709 .2374

INDIRECT EFFECT:

W_POIC -> InadCent -> QComp

Effect BootSE BootLLCI BootULCI

.0493 .0760 -.0978 .2059

INDIRECT EFFECT:

W_POIC -> GDcent -> InadCent -> QComp

P_GenBin Effect BootSE BootLLCI BootULCI

.0000 -.2618 .0721 -.4193 -.1349

1.0000 -.3413 .0916 -.5393 -.1785

Index of moderated mediation (difference between conditional indirect effects):

Index BootSE BootLLCI BootULCI

P_GenBin -.0795 .0329 -.1535 -.0261

---

*********************** ANALYSIS NOTES AND ERRORS ************************

Level of confidence for all confidence intervals in output:

95.0000

Number of bootstrap samples for percentile bootstrap confidence intervals:

10000

------ END MATRIX -----

**Additional Supplemental Analyses**

In a series of hierarchical linear regression analyses, we regressed each dependent variable onto: Step 1) dummy-coded participant gender (women = 0, men = 1), mean-centered proportion of household income earned by the female partner in each relationship (i.e., own proportion of income for female participants, partner proportion of income for male participants); Step 2) the two-way interaction between variables. We followed the procedures recommended by Aiken and West (1991) to decompose any observed interactions. Results are presented in Table 5 and described in the passages that follow. Once again, for brevity we will focus our interpretation on statistically significant results, though all results are presented in the Table.

**Relationship Quality*.*** As expected, as the proportion of income earned by the female partner in the participants’ marriage increased, relationship quality decreased In addition, men reported higher relationship quality than women in general.

**Gender Nonconformity.** Consistent with our hypotheses, all participants experienced stronger feelings of gender nonconformity as the proportion of income earned by the female member of the couple increased.

| **Table S3**  *Relationship quality reported by participants in heterosexual relationships in Study 3, as a function of participant gender and proportion of income earned by the female partner in the participant’s marriage* | | | | | | | |
| --- | --- | --- | --- | --- | --- | --- | --- |
| **Dependent Variable** | ***β*** | ***b*** | **95% CI** | ***t*** | ***p*** | **Δ*R*^2^** | ***f*^2^** |
|  |  |  |  |  |  |  |  |
| **Relationship Quality** |  |  |  |  |  |  |  |
| *Step 1* (*df* = 504) |  |  |  |  |  | .03*** | .03 |
| Gender | .13 | 0.27 | 0.08, 0.46 | 2.80 | .005 |  |  |
| Female POI | -.10 | -0.52 | -0.97, -0.07 | -2.26 | .024 |  |  |
| *Step 2* (*df* = 501) |  |  |  |  |  | .00 | .00 |
| Gender X Female POI | .02 | .14 | -0.77, 1.05 | 0.30 | .764 |  |  |
| **Gender Nonconformity** |  |  |  |  |  |  |  |
| *Step 1* (*df* = 505) |  |  |  |  |  | .04*** | .04 |
| Gender | .07 | 0.29 | -0.15, 0.62 | 1.67 | .097 |  |  |
| Female POI | .19 | 1.72 | 0.92, 2.51 | 4.23 | .000 |  |  |
| *Step 2* (*df* = 504) |  |  |  |  |  | .00 | .00 |
| Gender X Female POI | .02 | 0.34 | -1.28, 1.94 | 0.41 | .683 |  |  |
| **Feelings of Inadequacy** |  |  |  |  |  |  |  |
| *Step 1* (*df* = 505) |  |  |  |  |  | .02* | .02 |
| Gender | .06 | 0.20 | -0.08, 0.48 | 1.38 | .168 |  |  |
| Female POI | .12 | 0.89 | 0.22, 1.55 | 2.62 | .009 |  |  |
| *Step 2* (*df* = 504) |  |  |  |  |  | .01* | .01 |
| Gender X Female POI | .13 | 1.47 | 0.13, 2.82 | 2.16 | .031 |  |  |

*Note*. **p* < .05; ****p* < .001. POI = proportion of income earned

**Feelings of Inadequacy.** As expected, participants generally felt more inadequate as the proportion of income earned by the woman in their relationship increased. But this effect was moderated by gender in a manner that was consistent with our hypotheses concerning precarious manhood (see Figure 5). Women’s feelings of inadequacy did not vary directly as a function of the proportion of income they earned, *β* = .03, 95% CI [-0.62, 1.14], *t*(504) = 0.58, *p* = .563, but men felt more inadequate as the proportion of income earned by their female spouse increased, *β* = .23, 95% CI [0.72, 2.75], *t*(504) = 3.35, *p* < .001. However, these associations between breadwinner status and inadequacy do not tell the whole story concerning FBRs and gender threat because they do not account for gender nonconformity, and thus they do not reflect the totality of gender threat. Our next analyses will provide a better understanding of the links between breadwinner status, gender threat, and relationship quality.

**Figure S1**

*Feelings of inadequacy for heterosexual participants in Study 3, as a function of participant gender and the proportion of income earned by the female partner in the participant’s marriage*

*Note.* Results are graphed for participants in relationships where the proportion of income earned by the female partner in their marriage was one standard deviation below (i.e., woman earns 20%) or one standard deviation above (i.e. woman earns 60%) the sample mean.
